# Sub-cellular level resolution of common genetic variation in the photoreceptor layer identifies continuum between rare disease and common variation

**DOI:** 10.1371/journal.pgen.1010587

**Published:** 2023-02-27

**Authors:** Hannah Currant, Tomas W. Fitzgerald, Praveen J. Patel, Anthony P. Khawaja, UK Biobank Eye and Vision Consortium, Andrew R. Webster, Omar A. Mahroo, Ewan Birney

**Affiliations:** 1 European Molecular Biology Laboratory, European Bioinformatics Institute, Cambridge, United Kingdom; 2 Novo Nordisk Foundation Center for Protein Research, Faculty of Health and Medical Sciences, University of Copenhagen, Copenhagen, Denmark; 3 NIHR Biomedical Research Centre, Moorfields Eye Hospital NHS Foundation Trust and UCL Institute of Ophthalmology, London, United Kingdom; 4 Section of Ophthalmology, King’s College London, St Thomas’ Hospital Campus, London, United Kingdom; 5 Physiology, Development and Neuroscience, University of Cambridge, Cambridge, United Kingdom; Case Western Reserve University, UNITED STATES

## Abstract

Photoreceptor cells (PRCs) are the light-detecting cells of the retina. Such cells can be non-invasively imaged using optical coherence tomography (OCT) which is used in clinical settings to diagnose and monitor ocular diseases. Here we present the largest genome-wide association study of PRC morphology to date utilising quantitative phenotypes extracted from OCT images within the UK Biobank. We discovered 111 loci associated with the thickness of one or more of the PRC layers, many of which had prior associations to ocular phenotypes and pathologies, and 27 with no prior associations. We further identified 10 genes associated with PRC thickness through gene burden testing using exome data. In both cases there was a significant enrichment for genes involved in rare eye pathologies, in particular retinitis pigmentosa. There was evidence for an interaction effect between common genetic variants, *VSX2* involved in eye development and *PRPH2* known to be involved in retinal dystrophies. We further identified a number of genetic variants with a differential effect across the macular spatial field. Our results suggest a continuum between common and rare variation which impacts retinal structure, sometimes leading to disease.

## Author summary

The photoreceptor cells (PRCs) are found at the back of the retina and are responsible for detecting light and converting this to an electrical signal. Examination of these cells using optical coherence tomography (OCT) is often used to aid diagnosis and monitor several eye diseases including age-related macular degeneration and retinal dystrophies. Here we harnessed the large scale of the UK Biobank to study genetic effects on the thickness of the PRC layers, a measure extracted from the OCT images. We found a large number of common genetic variants associated with these measures, many of which are in or near genes known to be involved in eye biology or eye diseases. In particular we found many genetic variants associated with PRC thickness that are in or near genes known to be involved in a set of rare diseases called retinal dystrophies. We also found that the interaction of two of these genetic variants has an effect on the PRC layer thickness. Overall, we provided evidence for a relationship between common genetic variation, and both common and rare variation in retinal biology.

## Introduction

**Fig 1:**
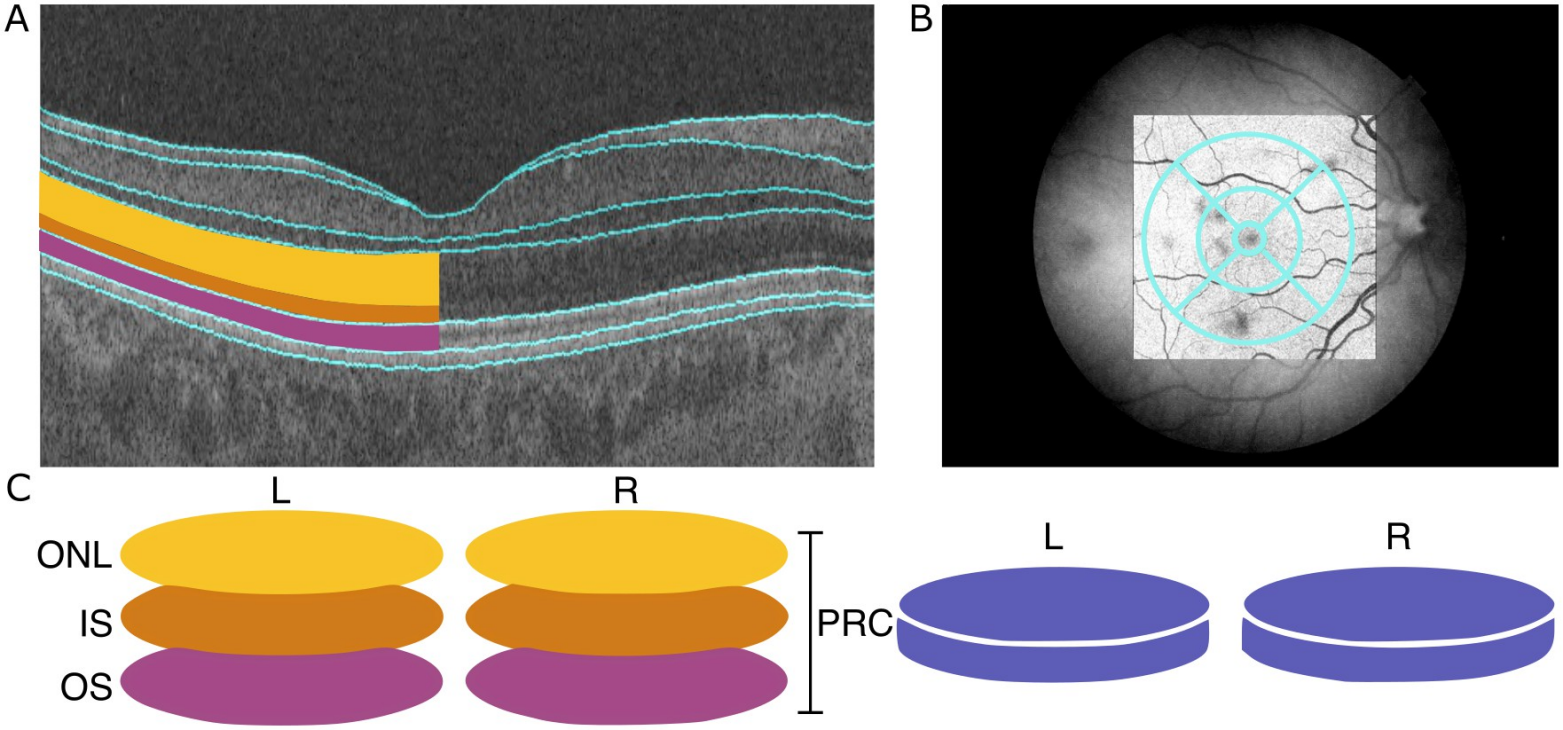
Data available from OCT images to describe morphology of the photoreceptor cell layer. (A) An optical coherence tomography (OCT) image of the cross-section of the retina. The layers are segmented, outlined in blue, by the Topcon advanced boundary software (TABS). On the left-hand side of the image, the PRC layers have been shaded: The outer nuclear layer (ONL) in yellow, the inner segment (IS) in orange and the outer segment (OS) in magenta. (B) The early treatment of diabetic retinopathy study (ETDRS) segmentation grid super imposed on a fundus image of the retina. The ETDRS grid is composed of nine segments arranged in a bulls-eye. (C) A schematic of the data utilised to describe PRC morphology as an input phenotype in genome-wide association studies (GWAS). The ONL, IS and OS, combined, comprise the photoreceptor cells (PRC).

The retina is a layered structure at the back of the eye, responsible for receiving light stimulus and converting it to neurological signal which is interpreted by the visual system of the brain. Each retinal layer comprises different cells that are responsible for particular stages of the signal conversion process. The central area of the retina is termed the macula, and has a valley like structure. At the centre of the macula, the bottom of the valley structure, is the fovea which is the area of the retina responsible for highest acuity vision [1]. The retina can be imaged using optical coherence tomography (OCT). OCT produces high resolution, three-dimensional representations of the retina in which the different retinal layers and macular structure can be identified (Fig 1A). OCT is a non-invasive imaging method commonly used in the clinic to diagnose and monitor a number of ophthalmic conditions [2].

The photoreceptor cells (PRCs) are found towards the back of the eye within the retina, backed by the retinal pigment epithelium layer (RPE) (Fig 1A), and are responsible for detecting light photons and generating electrical response through the process of phototransduction. They consist of two main types, the rods and cones. Rods are responsible for vision in low light but offer lower visual acuity and lack colour definition. Cones are active in higher light levels, and confer colour and high acuity vision. The cones are densely populated at the centre of the retina, the fovea, where there are no rods. Conversely, rods are most densely populated more peripherally, where cone density is low. The PRC layer can be further divided into three component intracellular layers; the outer nuclear layer (ONL), which comprises the cell body of the PRC, including the cell nucleus; the inner segment (IS), which contains both the PRC’s mitochondria and ribosomes required for photopigment assembly; and the outer segment (OS), which interfaces with the RPE and contains the stacked membranes in which photopigments are stored.

There are numerous pathologies, both rare and common, that affect the morphology of the PRCs. Notably, age-related macular degeneration (AMD) is defined by the accumulation of lipid deposits, known as drusen, under the RPE. This causes damage to the RPE and subsequently degradation of the PRCs [3]. AMD causes progressive and irreversible loss of visual function, and is a major cause of blindness in older people [4].

Many retinal dystrophies affect the photoreceptors [5]. These rare inherited disorders are due to highly penetrant mutations in genes expressed in PRCs or less commonly the RPE, resulting in retinal dysfunction with or without degeneration of the outer retinal layers. These can affect predominantly the central retina (macular dystrophies), the rod photoreceptors (rod-cone dystrophy or retinitis pigmentosa), or the cone photoreceptors (cone and cone-rod dystrophies). Though individually rare, taken together these diseases are a major cause of blindness in children and working age adults.

There have been previous studies on retinal morphology with known associations to age, sex, and ethnicity [6, 7]. Previous studies have further looked at the genetic variation underlying the overall retinal thickness [8] and the thickness of the inner retinal layers [9]. These studies identified loci with prior associations to general retinal development and ophthalmic disease.

In this paper, we utilise data within the UK Biobank to interrogate how genetic variation affects PRC morphology using quantitative phenotypes extracted from OCT images. The UK Biobank provides a large-scale rich resource of both genotypic and phenotypic data. We aim to identify genetic variants associated with quantitative variation in the morphology of the PRC layers and thus further our understanding of retinal development, biology and pathology. Here we conduct genome-wide association studies (GWAS) of the thickness of the component PRC layers, the ONL, IS and OS. Following meta-analysis of the three PRC layers, 111 loci are significantly associated with the thickness of one or more PRC layers. These loci include variants with prior associations to ophthalmic phenotypes, including ophthalmic diseases. 27 of the discovered SNPs were novel and had no prior associations to ocular or general phenotypes. Further analysis utilising the exome sequence data identified 10 genes associated with the thickness of one or more of the PRC layers. Several of these genes also had prior associations to ocular phenotypes. We further explored the genetic variation of the morphology of the retina at a higher resolution by considering the retinal layer thickness in different concentric areas of the macula. This identified a number of loci that appear to differentially affect PRC morphology across the retinal field. Interestingly, several of these loci have prior associations to retinal diseases whose pathological action bears similarity with the spatial variation in genetic effect on morphology. Additional analysis identified interaction between two of the common genetic variants, *VSX2* and *PRPH2*, affecting PRC thickness.

## Methods

### Ethics statement

The UK Biobank study was conducted with the approval of the North-West Research Ethics Committee (ref 06/MRE08/65), in accordance with the principles of the Declaration of Helsinki, and all participants gave written informed consent. The research presented here has been conducted using the UK Biobank Resource under Application Number 2112.

### UK Biobank data

The UK Biobank is a cohort comprising ∼500,000 individuals recruited through the NHS registry at age 40–69 from across the UK. Individuals were were not selected on the basis of having disease, resulting in a broad cross-section of the UK population. For all participants, a number of baseline physical measurements were taken and an extensive questionnaire completed comprising information on lifestyle, demographic and socioeconomic factors. Additionally, blood and urine samples were collected and further tests including a heel-bone ultrasound, bio-impedance, hand-grip strength, spirometry, blood pressure and several cognitive tests were performed. Each individual was further genotyped and exome sequenced. The participants also agreed to ongoing linkage of their medical records [10].

### Ophthalmic measurements and optical coherence tomography

A subset of the UK Biobank participants completed an eye examination. The measures collected included best corrected visual acuity using a logarithm of the minimum angle of resolution (logMAR) chart (Precision Vision, LaSalle, Illinois, USA). Visual acuity was measured with participants wearing their distance glasses at 4m, or at 1m if a participant was unable to read letters at 4m. Participants were asked to read from the top of the chart downwards, with the test terminated when two or more letters were read incorrectly. Further, refractive error was measured using a Tomey RC-5000 auto refraktometer (Tomey Corp., Nagoya, Japan) [11]. For each eye, up to 10 measurements were taken and the most reliable measure was automatically recorded. Intraocular pressure (IOP) was measured using an Reichert Ocular response Analyzer [12] from which corneal compensated IOP was calculated that accounts for rigidity of the cornea [13].

#### Optical coherence tomography imaging data

A further subset of 67,310 individuals underwent Spectral Domain Optical Coherence Tomography (SD-OCT) imaging. OCT imaging was conducted using the the Topcon 3D OCT1000 Mark II machine using 3-dimensional 6×6mm^2^ macular volume scan mode (512 A scans per B scan; 128 horizontal B scans). The imaging was completed on undilated eyes in a dark room following other eye measurements [13]. The right eye was consistently imaged first, and the majority of individuals had imaging repeated in their left eyes [12].

The images were processed using the industry standard Topcon advanced boundary software (TABS) [14]. This software performs automated retinal layer segmentation. It calculated thickness values for each of the retinal layers averaged across the macular field, as well as in each of the subfields of the early treatment of diabetic retinopathy study (ETDRS) grid [15]. The ETDRS is a nine-segment grid arranged as a bullseye and is commonly used during clinical assessment of the macula including the PRCs (Fig 1B).

### Quality control of genotypic and phenotypic data

**Fig 2:**
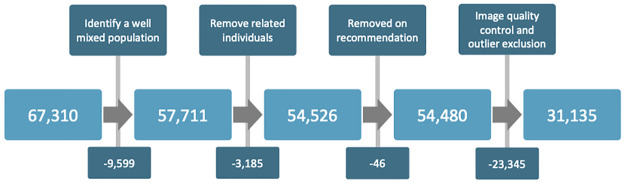
Cohort selection workflow. A schematic of the workflow applied during quality control of both genotypic and phenotypic data.

The most densely populated well-mixed population was selected for using principal component analysis applied to the genotype data. PCA was applied to a subset of SNPs following pruning based on linkage disequilibrium with a window size of 50kb, a step size of 1 and an r^2^ threshold of 0.8, using PLINK [16]. Further a minor allele frequency (MAF) threshold of 0.1 was applied. Individuals within a defined euclidean distance (1.45 × 10^-3^) of the mean of the selected population (CEU, Utah residents with Northern and Western European ancestry, and TSI, Toscana in Italia), as identified by comparison to the HapMap Phase III study [17] in PC1-PC2 space (PC1 = 7.52 × 10^-4^, PC2 = -4.66 × 10^-4^) were selected. This subset largely aligns with those that self-identify as White British in the UK Biobank ethnicity field. Individuals were further excluded if they were related to third degree or more [18]. Participants who were recommended for exclusion from genetic studies by the UK Biobank were removed from the dataset (Fig 2).

In addition to the genotypic quality control, rigorous quality control was applied to the phenotypic data. Exclusion/inclusion criteria were applied to the OCT images and the quantitative measures derived from them utilising methods previously implemented in Patel *et al*. (2016) [19]. In line with this method all participants with an OCT image quality score <45 were removed from the study. Further, individuals with values within the poorest 20% of the population in each of the OCT segmentation indicators were removed. These segmentation indicators include: Inner limiting membrane (ILM) indicator, a measure of the minimum localised edge strength around the ILM boundary across the entire OCT scan. ILM indicator is indicative of blinks, severe signal fading, and segmentation errors; Valid count, used to identify significant clipping in the z-axis of the OCT scan; Minimum motion correlation, maximum motion delta and maximum motion factor, all of which utilise the nerve fibre layer and total retinal thickness to calculate Pearson correlation and absolute differences between the thickness values from each set of consecutive B-scans. The lowest correlation and highest absolute difference in a scan define the resulting indicator values. These values identify blinks, eye motion artefacts and segmentation failures. It should be noted that the image quality score and segmentation indicators are often correlated with one another. Finally individuals with outlier values of refractive error were removed from the study. Outlier refractive error scores were defined as values lying outside one standard deviation of 1.5 times the inter-quartile range from the median. The final dataset included 31,135 individuals.

### Genome-wide association studies

GWAS were implemented using an additive linear model in BGENIE [18]. The mean thickness of each component PRC layer across the ETDRS grid was used as the input phenotype (Fig 1C). Eye-specific covariates including image quality measures obtained from the OCT machine (listed above) and refractive error (calculated as *spherical* *error* + 0.5 × *cylindrical* *error*) were regressed out of thickness values for the left and right eye separately before a mean was made across the two eyes. Covariates including age, weight, sex, height, OCT machine ID and the first 20 genotype PCs were used in the model. A genome-wide significance threshold of P <5 × 10^-8^ was used. LD-score regression was implemented using LD SCore v1.0 [20] to obtain estimates of genomic inflation and heritability of traits.

#### Associated variant discovery set

The summary statistics from the GWAS of the ONL, IS and OS were meta-analysed using MTAG [21]. The resulting lowest p-value of the three meta-analysed phenotypes was considered the meta p-value. The meta-analysed PRC results were filtered using GCTA Conditional and Joint Analysis (COJO) [22] to select for independent SNPs that were more than 10Mb apart. Further filtering was applied and loci within 1.5Mb of one another were labelled as being in the same locus as indicated in the results tables where loci considered to be within the same locus are shaded the same colour, alternating blue and white. A plot of a magnified locus of interest was generated using LocusZoom [23].

### Exome analysis

We performed gene burden testing using the UK Biobank 200,000 whole exome release to search for genes that showed an increased level of rare loss of function (LoF) or rare missense variants that were predicted as deleterious and probably damaging. Briefly, we ran the Ensembl variant effect predictor (VEP) [24] on the multi sample VCF from the 200K UK Biobank release to allow us to classify variants and assign their consequence. We defined high confidence LoF variants using the VEP plugin LOFTEE and selected missense variants using SIFT “deleterious” and Polyphen “probably_damaging” flags, all variants were filtered to have a minor allele frequency (MAF) of less than 1% prior to collapsing into gene based genotypes for burden testing. To define gene level genotypes we used the standard collapsing method where we considered all variants within the gene for each sample separately and defined zero, one and two (0,1,2) genotypes based on: zero (genotype 0) no rare variant of the given type present in the gene; one (genotype 1) more than zero rare variants of the given type present in the gene but all are in the heterozygous state; and two (genotype 2) if any of the rare variants of the given type where in the homozygous state. It is worth noting that by using this approach we do not account for compound heterozygous mutations or the overall variant load within individual genes but it does allow us to interrogate potential rare variant associations to the trait of interest at the gene level. To perform the association testing we treated the gene level genotypes for rare LoF and potentially damaging missense variants as standard genotypes within a linear model with covariates against the OCT derived PRC layers. This used a cohort consisting of a subset of the individuals included in the GWAS who also had exome sequencing data available. The covariates included within the models and the overall sample selection was exactly the same as for the common SNP GWAS analysis. A significance threshold of P <5 × 10^-5^ was used.

### Annotation of variants and geneset enrichment

Significant variants were manually annotated with associated gene and any prior ophthalmic or general phenotypic associations using ENSEMBL [25] and Open Targets Genetics platform [26]. Geneset and tissue enrichment analysis was conducted using DEPICT [27].

### Comparison of genetic effect on retinal sub-fields

**Fig 3:**
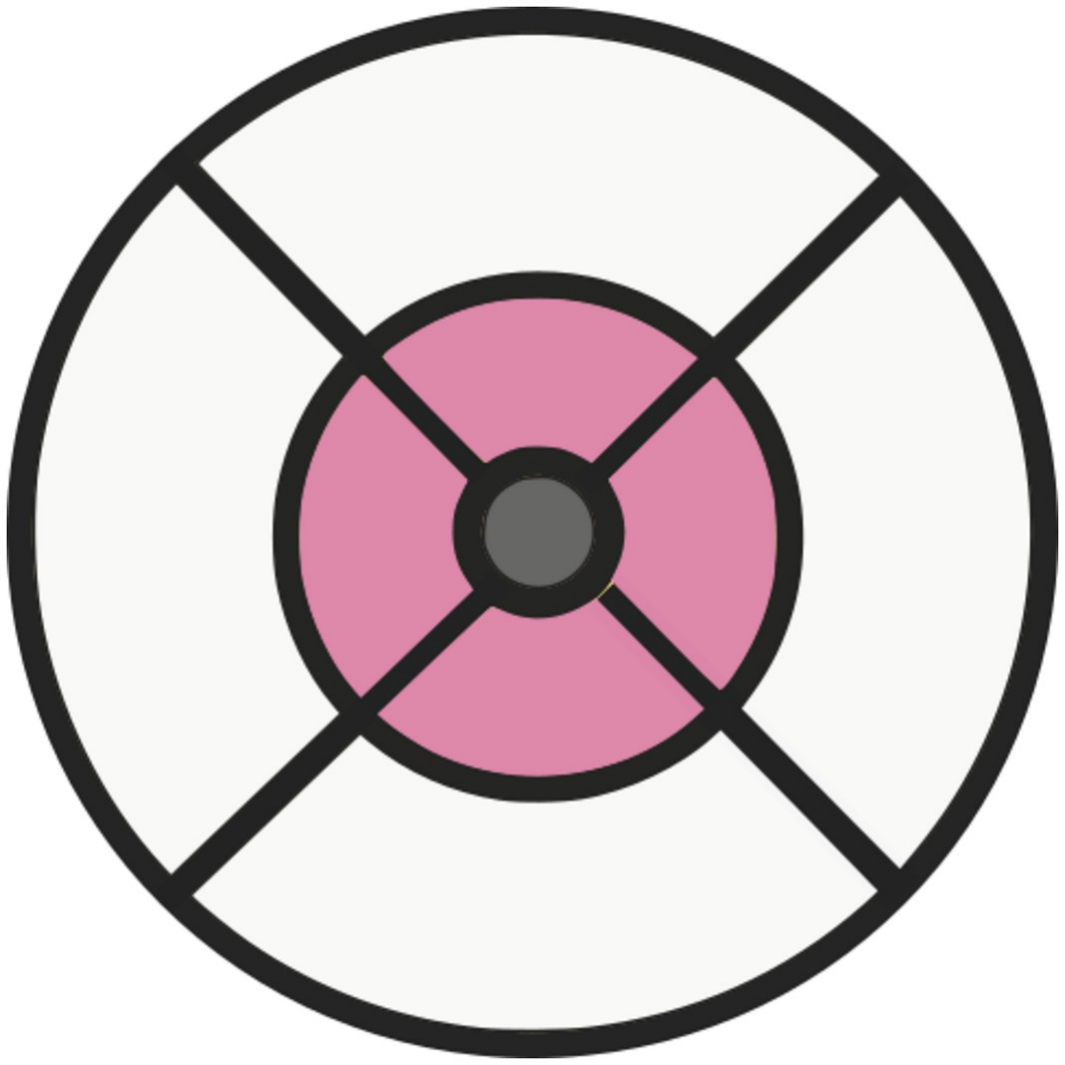
Comparison of concentric rings defined by the ETDRS grid. The macula divided into three concentric fields for comparison utilising the ETDRS grid. The peripheral field is shaded white, the intermediate field is shaded pink and the foveal field is shaded grey.

The foveal, intermediate and peripheral fields of the ETDRS grid were considered separately (Fig 3). A mean thickness of each of the PRC layers was calculated across each of these fields. A GWAS was then performed for each of these phenotypes. Eye specific covariates were regressed out of the individual eye measurements prior to a mean being made across left and right eyes, as described above. The same covariates were used in these GWAS as were in the simple phenotype GWAS. The GWAS were performed using BEGENIE [18].

To compare the genetic effect of each SNP on the different subfields, each retinal layer was considered at a time. For each PRC layer, comparisons were made between the genetic effect on: the foveal field compared to the intermediate field; the foveal field compared to the peripheral field; and the intermediate field compared to the peripheral field. For each comparison, SNPs were selected that were significantly associated with either of the two phenotypes being considered. A z-score was calculated for each of the SNPs comparing the genetic effect size for the two phenotypes. The z-score was defined as:

$$Z=\frac{|\beta_{0}-\beta_{1}|}{\sqrt{SE_{0}^{2}+SE_{1}^{2}}}$$

where *β* is the genetic effect size and *SE* is the associated standard error. P-values were calculated for each of the z-scores and SNPs selected that were significant following Bonferroni correction. The list of SNPs was clumped into loci based on linkage disequilibrium values. Each variant was annotated with all SNPs with r^2^>0.05 in a 1000kb window using PLINK [16] and the locus with the lowest p-value in each window selected.

### Visualisation of spatial genetic effect

A linear model was assessed of the effect of each of the loci identified as differentially affecting the concentric retinal fields on the thickness of each of the PRC layers in each of the nine segments in the ETDRS grid. The model included the same covariates as the GWAS of average retinal thickness and the effect size was plotted back into the ETDRS grid space.

### Interaction models

Linear models were used to explore the presence of genetic interaction affecting the thickness of the three PRC layers. The same covariates were used as those used in the GWAS. Significance values were corrected for multiple testing using Benjamini and Hochberg correction and a false discovery threshold of < 0.15 was used.

## Results

**Fig 4:**
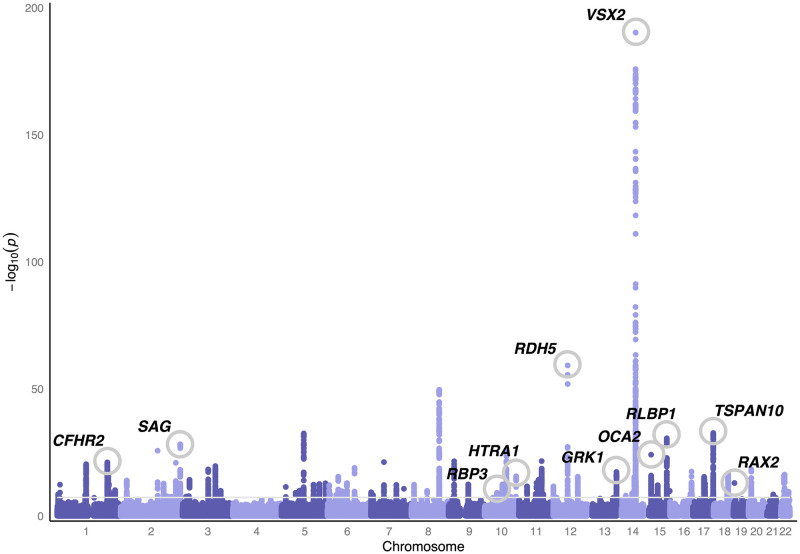
Genome-wide association study of photoreceptor cell layer thickness phenotypes. Manhattan plot of the photoreceptor cell layer thickness phenotype GWAS p-values. These result from meta-analysis using MTAG [21] of the summary statistics from GWAS of the thickness of the outer nuclear layer (ONL), inner segment (IS) and outer segment (OS). Variants are considered significantly associated if they reach genome wide significance (P <5 × 10^-8^). Loci of interest, selected through manual labelling by ophthalmic experts, are circled and labelled.

Initially we conducted three genome wide association studies (GWAS), one for the thickness of each component PRC layer—the ONL, IS and OS—averaged across both the ETDRS grid and across both left and right eyes (Fig 1C). This is analogous to many clinical uses of macular layer thicknesses. Quality control was applied to the OCT image data consistent with previous work [9, 19] and we used a well established method to select the European-associated PCA cluster using genetic data, which aims to minimise variation in non-genetic factors and genetic factors (See Methods). This grouping predominantly self identifies as White British in the UK Biobank ethnicity survey (though importantly many people who self-identify as White British in UK Biobank are not in this PCA-defined cluster). The resulting subset of samples that passed both image and genetic filters comprised of 31,135 individuals (S1 Table). The three GWAS (one for each component layer of the PRC), each identified a number of associated loci using the established genome-wide significance level of P <5 × 10^-8^ threshold. Following calculation of the linkage disequilibrium score for each of the three GWAS, there was minimal evidence for inflation due to residual population structure (S1 Fig and S2 Table). SNP heritability of the thickness of each of the PRC layers was calculated: ONL h^2^ = 0.39, IS h^2^ = 0.18, and OS h^2^ = 0.21. Unsurprisingly we observed numerous loci that were significantly associated with the thickness of more than one of the PRC layers, and so we meta-analysed results using MTAG [21]. Following selection of independent loci using COJO [22], there were 111 loci significantly associated with the meta-analysed PRC layers’ thickness (Fig 4 and S3 and S4 Tables). 27 of the discovered genetic variants were novel with no prior associations to ocular or general phenotypes.

**Fig 5:**
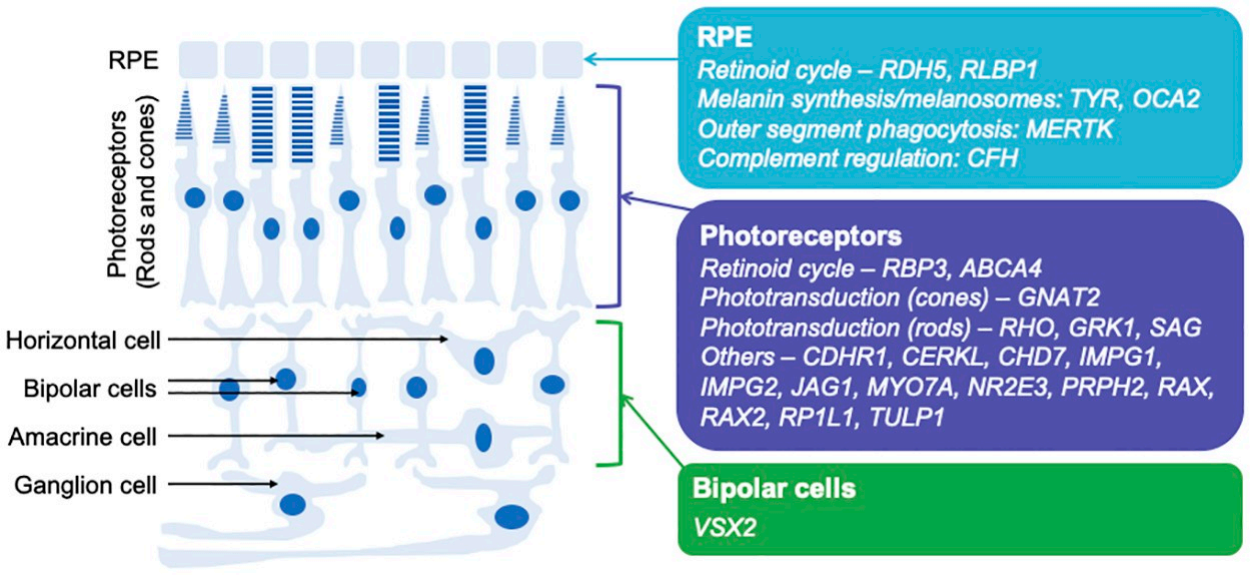
Schematic of retina showing main cell types with sites of expression (in mature human retina) of many of the genes associated with rare monogenic ocular disease found during genetic discovery (S5 Table). Some genes are also expressed significantly in other cell types not shown (e.g. *RLBP1* is expressed also in Muller cells). Also, some genes (including those not shown within this figure) have a wider role in development, but expression is more restricted in adults (e.g. *VSX2* is expressed in retinal progenitor cells in development, but mainly in bipolar cells in the mature retina).

We immediately noticed that many of these loci were close to well known genes associated with rare ocular diseases (Fig 5 and S5 Table). 17 of the 111 loci overlap with a known rare retinal disease locus, which is highly unlikely to occur by chance (P <2.2 × 10^-16^ hypergeometric distribution).

We further leveraged the initial release of the UK Biobank exome dataset of 247,000 individuals, complementing the common variation GWAS with a rare variation gene burden test on the same phenotypes. We considered both strict loss of function mutations and overall missense mutations as two separate tests applied to each layer. Although there were no strong rare variant hits, setting a more liberal significance threshold (P <5 × 10^-5^)—consistent with other exome wide burden tests—we found 10 significant loci (S6 Table and S2 Fig). Three of these loci overlap with rare eye disease, again unlikely to have been observed by chance (P = 2.65 × 10^-6^ by hypergeometric distribution).

Overall between rare and common discovery we have found over 100 loci associated with PRC thickness in some manner, and these continuous quantitative phenotype associations have significant overlap to known rare ocular disease genes.

### Selected loci of interest

**Fig 6:**
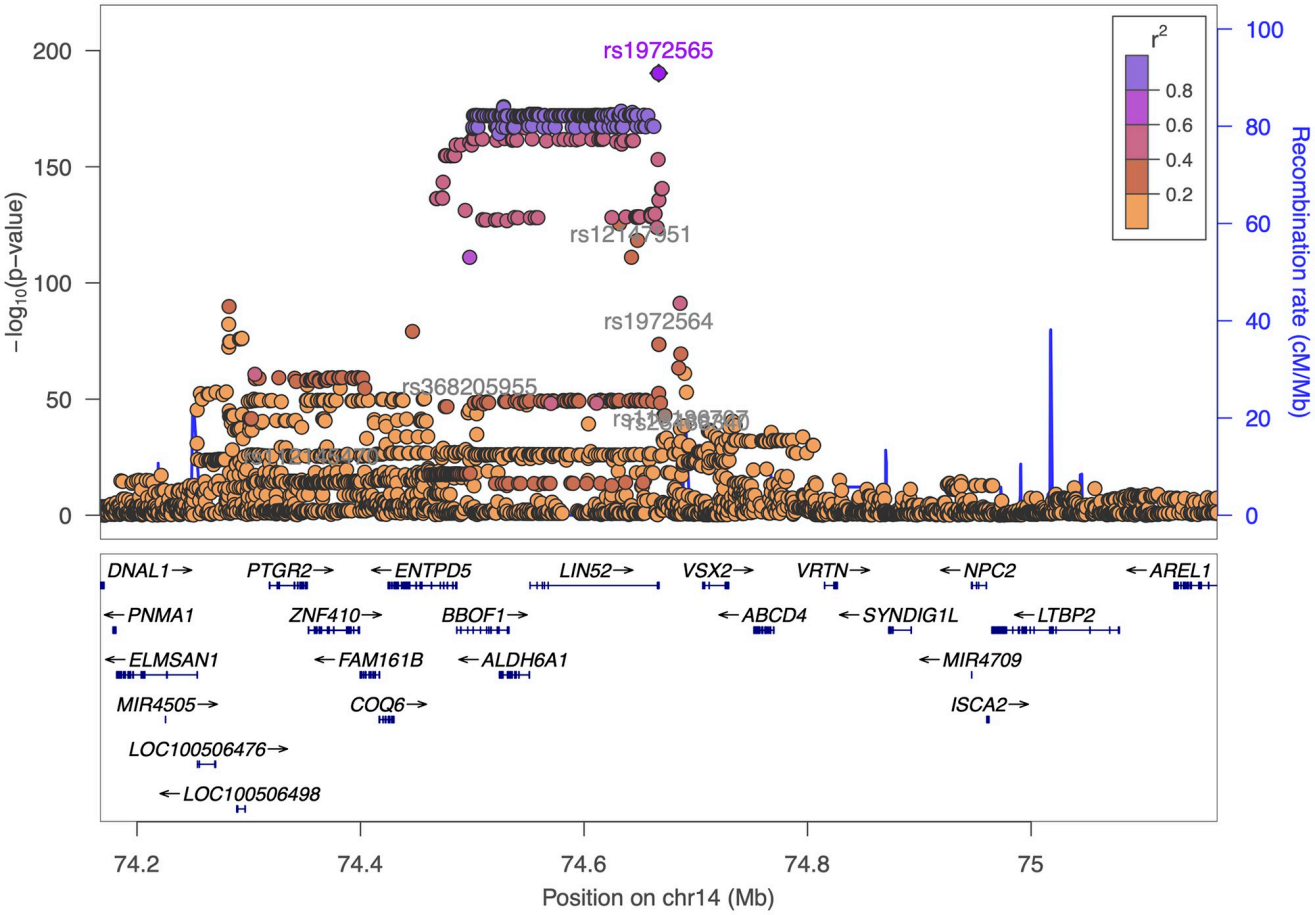
Magnification of the *VSX2* locus. Association of the locus on chromosome 14 centred on the genetic variant rs1972565 (highlighted in purple). Other genetic variants found significantly associated with PRC thickness are labelled in grey.

Of these genetic variants, the locus with the smallest p-value and with a reasonably large effect size for a single locus was *VSX2*. *VSX2* is a homeobox transcription factor that has been found expressed in the developing retina of humans, mice and zebrafish [28]. The gene is known to have an important role in ocular size, with pathogenic variants leading to microphthalmia [28]. This locus showed allelic heterogeneity in the GWAS results with the most significant genetic variant being rs1972565 (P = 5.23 × 10^-191^) whilst COJO analysis retained 4 other SNPs across the *VSX2* locus. In addition, there are SNPs in nearby genes which could be long range regulatory SNPs for *VSX2* rather than the closest genes we have assigned this to (Fig 6). Interestingly in previous work we identified genetic variants in neighbouring loci that had a statistically significant effect on the thickness of the inner retinal layers [9]. All this evidence points towards the *VSX2* locus having multiple alleles which affect different aspects of eye development.

Further there were several loci found associated with PRC layer thickness that are known to be involved in the retinoid cycle, specifically *RDH5*, *RLBP1* and *RBP3*. Each of these genes have a known role in the retinoid cycle and are associated with rare inherited retinal diseases. *RDH5* (rs3138142, P = 5.58 × 10 ^-60^) encodes 11-*cis* retinol dehydrogenase. The specific SNP in *RDH5* has previous associations with a number of ophthalmic phenotypes including the age one started wearing glasses, cataract and myopia. Pathogenic variants in this gene are known to cause fundus albipunctatus, a retinal dystrophy and form of night blindness that primarily affects the rods [29, 30]. *RLBP1* (rs3825991, P = 2.70 × 10^-31^) encodes the cellular retinaldehyde binding protein, a transporter of 11-*cis* retinal [31, 32]. Mutations in this gene lead to a number of different retinal dystrophies [33]. *RBP3* (rs111245635, P = 8.41 × 10^-10^) encodes the interphotoreceptor retinoid-binding protein which has a role in transporting retinoids between the RPE and the photoreceptor cells [34]. Additionally, rare variants within *RPB3* cause recessive retinal dystrophy and high myopia [35]. Much of the retinoid cycle occurs in the supporting RPE layer, for example *RDH5* is almost exclusively expressed in this layer [36], showing that one does not need gene expression in a cell type to impact its structure.

Two loci, *SAG* (rs7564805, P = 6.16 × 10^-29^) and *GRK1* (rs9796234, P = 4.69 × 10^-18^), were found associated with PRC layer thickness and these genes have known involvement in the rare autosomal recessive Oguchi disease. *SAG* encodes arrestin and *GRK1* encodes rhodopsin kinase both involved in the rhodopsin signalling cascade. Studies have also found changes in the microstructure of the retinal layers in Oguchi disease patients [37, 38]. The presence of common variation in these genes suggests that there might be functional differences in rod physiology due to genetics present at the population level as well as rare genetics.

Further, several variants were found in the large heterogenous *NPLOC4-TSPAN10* locus, and both rare and common variants were discovered in the *OCA2* locus. Variants in these loci have previously been found associated with foveal structure [9] suggesting that overall retinal development has an impact on the PRC layer. Additionally the *NPLOC4-TSPAN10* locus is associated with strabismus, the abnormal alignment of the eyes [39]. Strabismus is known to be caused by defects in the extraocular muscles, cranial nerves, refractive error and visual cortex processing. Our finding suggests there may be a role of retinal structure in strabismus which warrants further investigation utilising appropriate clinical datasets.

We also note that several loci significantly associated with the PRC layer thicknesses had prior associations to AMD. These included *CFHR2* (rs410895, P = 8.19 × 10^22^), *HTRA1* (rs60401382, P = 2.43 × 10^-16^) and *RAX2* (rs76076446, P = 1.07 × 10^-13^). AMD is a disease characterised by the atrophy of PRCs caused by the accumulation of lipid deposits in the RPE. Extensive recent work has previously been completed to study the relationship between PRC thickness and AMD [40, 41].

To further explore the overall biological pathways underlying PRC morphology, we used DEPICT [27] to perform gene set and tissue enrichment analysis. When applied to the loci associated with PRC thickness, numerous genesets reached statistical significance following false discovery rate (FDR) correction (S7 Table). These include ocular-associated genesets such as *abnormal lens morphology*, *microphthalmia*, *disorganized retinal layers* and *abnormal ocular fundus morphology* amongst others. Additionally, tissue enrichment analysis of the loci associated with PRC thickness resulted in significant associations with *retina* and *eye* tissue (S8 Table).

Additionally, there were a number of genes identified in the gene-burden test analysis with interesting prior associations. These include *OCA2*, also significantly associated in our GWAS analysis, which has a prior association to oculocutaneous albinism. Individuals with this condition are characterised by foveal hypoplasia, the absence of the formation of the foveal pit. Further, three genes, *ABCA4*, *MYO7A* and *NR2E3*, are shown to have an association to PRC thickness and have a prior association with retinal dystrophies. *ABCA4* encodes ATP-binding cassette sub-family A member 4, a protein expressed almost exclusively in the PRCs. *ABCA4* is the gene most frequently implicated in monogenic retinal dystrophies [5, 42]. Biallelic pathogenic variants are the most common cause of of juvenile macular degeneration (Stargardt disease) and can also give rise to a cone-rod dystrophy. *MYO7A* encodes myosin VIIA, a protein found in inner ear and retina. Mutations in *MYO7A* are known to cause Type 1 Usher Syndrome, a condition characterised by deafness and retinal degeneration [43]. *NR2E3* encodes a nuclear receptor transcription factor that activates rod development and represses cone development [44]. Mutations in this gene are also associated with retinitis pigmentosa in addition to enhanced S-cone syndrome [45].

### Fine grained topography of genetic variation

**Fig 7:**
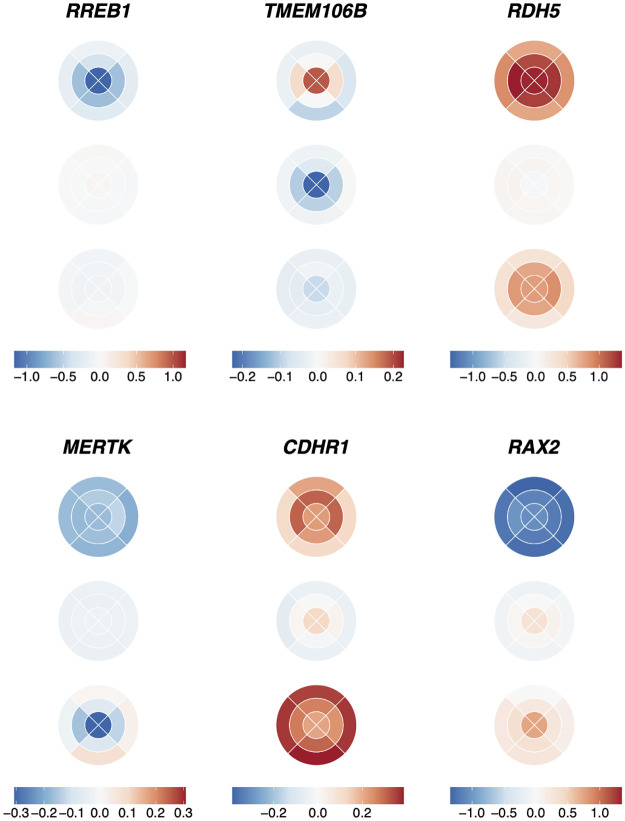
Effect size of selected SNPs on ETDRS grid. The figure shows the effect size of the SNPs (shown by a blue-red gradient) with differential effect on the concentric fields of the retina, on the nine different segments of the ETDRS grid across the three PRC layers listed from top to bottom: ONL, IS and OS. Six loci are visualised: *RREB1* (rs75757892), *TMEM106B* (rs13237518), *RDH5* (rs3138142), *MERTK* (rs869016), *CDHR1* (rs55798570), and *RAX2* (rs76076446). Effect sizes are scaled for each genetic locus individually.

In addition to the clinically utilised measure of the mean PRC thickness taken across the ETDRS grid, TABS outputs the thickness of each of the PRC layers within each of the nine ETDRS subsections. To capitalise on this increased dimensionality of data, we explored how genetic variation affects PRC morphology in the different areas of the ETDRS segmentation grid. We chose to consider the macular field split into three concentric fields which align with the topography of the valley-like macula (Fig 3). A mean thickness across each of these concentric fields—the foveal, intermediate and peripheral fields—was taken for each of the PRC layers. These phenotypes were used as an input to GWAS and the SNPs which passed the significance threshold of 5 × 10^-8^ were taken forward for further analysis. Z-score analysis was applied to the resulting effect size to identify loci with significantly different effect on thickness of the different concentric fields of each PRC layer. Numerous (32) loci were found in each layer that affect the thickness of the concentric fields differentially (Fig 7 and S3 Fig).

The locus showing the most significant difference in effect on the thickness of the ONL across the concentric fields was *RREB1* (rs75757892) which had a differential effect on the foveal field (effect size = -1.12) compared to both the intermediate field (effect size = -0.55) and peripheral field (effect size = -0.20). *RREB1* encodes a zinc finger transcription factor, the Ras-responsive element binding protein 1 [46]. Genetic variants at *RREB1* have prior associations to various ocular phenotypes including the age one starts wearing glasses and spherical power.

The locus showing the most significantly different genetic effect on the IS thickness across the macula was *TMEM106B* (rs13237518) which had a significant difference between the foveal field (effect size = -0.23), and the peripheral field (effect size = -0.02). *TMEM106B* is known to be involved in frontotemporal lobar degeneration, a neurodegenerative disease [47]. There is growing interest in the use of OCT derived retinal thickness measures as biomarkers for neurodegenerative diseases [48, 49] making this finding of potential interest.

The locus with the most significantly different genetic effect on the thickness of the OS was *RDH5* also found in the initial meta-GWAS. *RDH5* (rs3138142) shows a significantly different effect on the thickness in the peripheral field (effect size = 0.81) compared to both the foveal field (effect size = 1.37) and the intermediate field (effect size = 1.28). As described above *RDH5* encodes 11-*cis* retinol dehydrogenase, an enzyme that is part of the retinoid cycle. It has been previously associated with the retinal dystrophy, fundus albipunctatus.

Notably several further loci were discovered to differentially affect the concentric fields of the retina that had prior associations with retinal dystrophies. These include *MERTK* (rs869016) which shows a significant difference of effect across the concentric fields of the OS. It has a significantly larger effect size on the fovea (effect size = -0.31) as compared to the peripheral field (effect size = 0.02). *MERTK* encodes MER proto-oncogene, tyrosine kinase, a transmembrane protein localised to the RPE. *MERTK* has a known role in the phagocytosis of the OS by the RPE. Mutations in *MERTK* have been associated with retinitis pigmentosa and early onset retinal dystrophies [50].

Additionally *CDHR1* (rs55798570) shows a significantly different effect size on the thickness of the IS in the foveal field (effect size = 0.13) compared to both the intermediate field (effect size = 0.002) and the peripheral field (effect size = -0.06). *CDHR1* encodes cadherin-related family member 1, a cell adhesion protein that localises to the junction of the IS and OS. Genetic variants in *CDHR1* have been associated with a number of inherited retinal dystrophies including retinitis pigmentosa and cone-rod dystrophy [51].

Further *RAX2* (rs76076446), shows a different effect on the thickness of the IS at the fovea (effect size = 0.31) compared to the peripheral field (effect size = -0.09). *RAX2* encodes retina and anterior neural fold homeobox 2, which has a known role in eye development. Pathological variants in this locus are known to cause cone and rod dysfunction and retinitis pigmentosa, a rod-cone disease [52].

Each of these retinal dystrophies have distinct and sometimes opposing spatio-temporal patterns in which they affect the rods and cones. Some dystrophies, including retinitis pigmentosa, affect the rods first, with the cones affected later. Patients have problems with night vision, then peripheral vision, and finally central vision. Fundus albipunctatus also affects rod function, whilst cones are usually spared. Others such as cone-rod dystrophy affect the cones first and the rods afterwards. Cones are found at the highest density at the fovea, whilst rod density peaks more peripherally. There appears to be a parallel between the spatial genetic effects of these loci on thickness of the PRC and the organisation of PRCs affected by the diseases associated with these loci.

### Genetic interactions

As there are both clear common biological pathways associated with multiple discovered variants, and overlaps with known rare disease loci we wanted to explore whether genetic interactions were affecting the retinal phenotype. Genetic interactions of common variation are challenging to discover and characterise due to the large space of possible interactions. To restrict our space we considered interactions in the following categories: (1) Interactions of the developmentally important *VSX2* locus internally and with all other common genetic variants; (2) Interactions between the Retinoid cycle loci; (3) Interactions of the Oguchi syndrome loci; (4) Interaction of all loci with genetically defined sex; and (5) Interactions between rare burden levels in genes and nearby common SNPs.

Overall we do not find widespread interaction terms that would pass multiple testing criteria. There was no statistically significant interaction term between the genetic variants with prior association to Oguchi disease, or between those with a prior association with the retinoid cycle. Additionally, there was no evidence of an interaction between any of the 111 identified loci and genetically defined sex. There was no evidence for interactions between rare burden levels in genes and nearby common SNPs; however this analysis was limited by the sample size of individuals with heterozygous alternative allelic states. Expansion of this analysis in a cohort containing a higher proportion of individuals with rare disease phenotypes would have more power to identify interactions.

**Fig 8:**
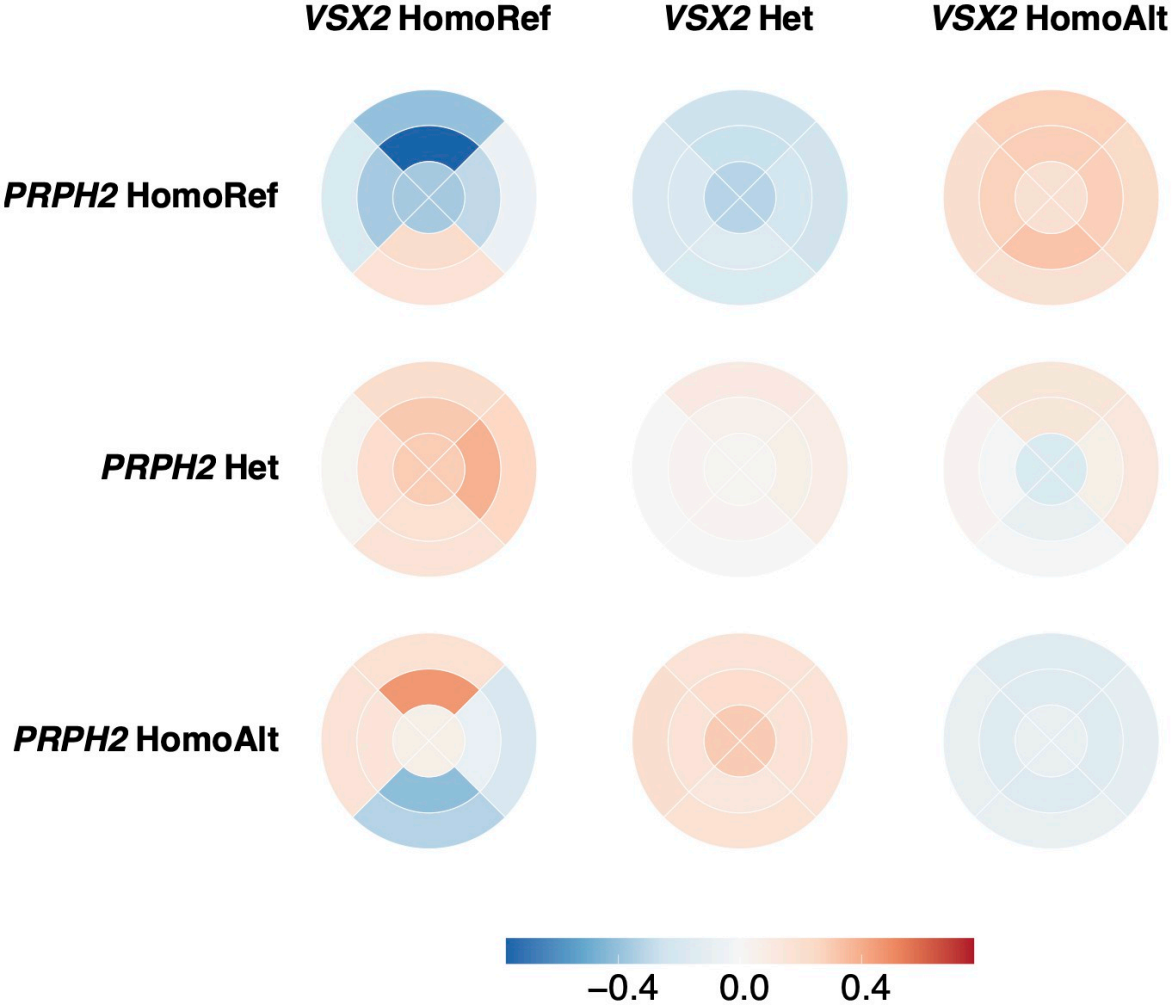
Normalised thickness of ONL stratified by *VSX2* and *PRPH2* genotype. The mean thickness of the ONL at each of the nine segments of the ETDRS grid across individuals stratified by their genotype at *VSX2* and *PRPH2*. The thickness is mean normalised per *VSX2* genotype.

However, following Benjamini and Hochberg correction for multiple testing, and using a False Discovery threshold level of < 0.15, four variants show an interaction with the lead *VSX2* SNP, rs1972565 in relation to their effect on ONL thickness. Two of the four variants (rs112145470 and rs28488340) are within the same locus as *VSX2*, consistent with the already noted allelic hetreogenity at this locus. The other two loci are *DYNLRB2* (rs7206532) and *PRPH2* (rs375435). *DYNLRB2* is expressed in retinal Muller cells and astrocytes, and encodes dyenin light chain roadblock type 2. Mutations in this gene are associated with deafness and hepatocellular cancer. *PRPH2* encodes peripherin 2 which is localised to the photoreceptors. Mutations in this gene are known to result in forms of retinal dystrophy and macular degeneration [53]. Upon stratification of the population by their genotype at *VSX2* and *PRPH2*, a change in ONL thickness can be observed, specifically between *PRPH2* homozygous reference and homozygous alternative genotype in those with the *VSX2* homozygous reference genotype (Fig 8 and S4 Fig). In individuals with *PRPH2* homozygous reference, a thinner ONL is found in the superior segments of the macula, and thicker ONL in the inferior segments. In individuals with *PRPH2* homozygous alternative, the inverse pattern is seen. This is of particular interest as there is often a difference in PRC degeneration across the macula equator in retinal dystrophies. *PRPH2* has a documented spectrum of penetrance and associated disease phenotypes [54, 55]. This finding suggests one of the possible mechanisms by which the effects of variants at the *PRPH2* locus are modified is via the genotype at the *VSX2* locus.

## Discussion

Here we have performed the largest and most detailed GWAS and rare variation association of PRC layer thickness to date, leveraging the high-dimensional spatial resolution available within retinal OCT. We initially explored a simple phenotype of the mean thickness of each of the PRC layers—the ONL, IS and OS—across the entire macula field as defined by the ETDRS grid. These phenotypes are advantageous in that they are aligned with those used in clinical practice. Using these phenotypes, we identified 111 independent loci significantly associated with one or more of the component PRC layers following GWAS meta-analysis and 10 loci associated with rare variation following gene burden testing. Whilst 27 of the discovered genetic variants were novel with no prior associations, many of the loci identified here had prior associations to ocular phenotypes and the loci collectively were enriched for known rare ocular diseases.

The collection of rare disease loci discovered in this study of common, physiologically healthy individuals without obvious eye diseases shows the continuum between common and rare variation, and rare variation which leads to disease. In this study we explicitly looked for interactions between common variation at different loci and common and rare variation at the same loci. We found several relatively weak interactions between a SNP near the well known eye development gene, *VSX2*, and two SNPs within this locus as well as two SNPs near *PRPH2* and *DYNLRB2*. No evidence was found for an interaction between common and rare genetic variants but this was partially due to limitations of the dataset and its absence of adequate numbers of individuals with rare variation and subsequent lack of statistical power.

Genetic discovery also found sets of loci associated with PRC thickness with prior associations to different ocular phenotypes. Four loci (*GNAT2*, *SAG*, *GRK1* and *RHO*) have a prior association to phototransduction, the generation of an electrical response to light in the PRC. Further three variants were found in or near genes (*RDH5*, *RLBP1* and *RBP3*) with prior associations to the retinoid cycle, the process by which the chromophore is regenerated following photoisomerisation by light. In both cases, this finding suggests a relationship between the form and function of these cells which may warrant further investigation. Whilst it might be intuitive that genes encoding structural and matrix proteins are associated with photoreceptor layer thicknesses, it is interesting that genes encoding proteins involved in bringing about or shaping the photoreceptor electrical light response to light are also implicated, potentially suggesting a role for visual signalling in development or maintenance of outer retinal structure.

There were several loci associated with specific disease. For example both *SAG* and *GRK1* in addition to an association with PRC thickness have a known involvement in Oguchi disease, a rare autosomal eye condition which causes visual impairment. As well as the overlap between rare genetic eye disease and these common variants affecting retinal structure, there are some interesting, less expected findings. For example, three loci—*MYEOV* (rs10737153), *TNS1* (rs201030469) and *FGFR2* (rs17102399)—have impacts on both PRC layer thickness and risk of diagnosis for breast cancer. Additionally *TMEM106B*, which had a particularly notable spatial genetic effect, has a known association to a form of dementia. In both cases, the link between morphology and disease suggests the possibility of using OCT derived PRC morphology measures as biomarkers for complex diseases in the future. Additional datasets enriched for individuals with these diseases with OCT data will be required to further explore these concepts.

It is also worth noting that there were 27 genetic variants discovered to be associated with PRC thickness that had no prior associations to phenotypes, ocular nor general. Some of these variants are at loci with known involvement in ocular pathology or development. These include rs111163508 at *RHO*, rs9796234 at *GRK1* and numerous variants including rs1972565 at *VSX2*, all discussed previously. However there are also a number of variants discovered that do not have prior associations and are not at loci with an obvious involvement in retinal biology. For example, rs1372613 at *LINS1* which encodes a protein involved in modulating the Wnt signalling pathway. Mutations in this gene have been seen in individuals with neurodevelopmental disorders [56]. This variant and others highlighted in S3 Table as having no prior phenotypic associations warrant further investigation into their involvement in retinal biology.

Several genes were identified through more than one of our three analyses—GWAS, exome analysis and differential concentric analysis (S5 Fig). For example *OCA2* was found to be significantly associated with PRC thickness in the GWAS and in the exome gene burden analysis. This suggests that *OCA2* may act through numerous genetic mechanisms, both that caused by common and rare variation. This is in-keeping with our previous discussion of the spectrum of variation in morphological variation, from the more extreme and pathological, to the common. There were also 10 genes that were found in both the GWAS and the differential genetic analysis of variation in morphology across the macular field. This could suggest that several genes have both broad control of morphological development of the macula but further fine-grain control that specifies detailed morphology across the macular field. It could also suggest that the effect of these genetic variants on certain areas of the macula is large enough to affect the average thickness of the PRC layers and hence be further discovered in the GWAS analysis. On the other hand, there was no crossover in the genetic discovery of the exome analysis and the differential concentric analysis. This could be because the differences in morphology caused by genetic variation captured by the differential concentric analysis are subtle, whereas the phenotypic variation expected as a result of rare variation captured by the exome analysis would be larger.

There are several limitations to acknowledge within our study. Firstly, the results within the GWAS have not been replicated in an independent dataset. This is largely due to the absence of an appropriate and comparable dataset that contains coupled genotypic data and quantitative measures of PRC morphology. In the future this study should be repeated to confirm the associations discovered here. In particular this would have great value for the novel genetic variants discovered. Secondly, the exome data used within the gene burden testing was unphased meaning we were unable to differentiate between *cis-* and *trans-* compound heterozygotes. This approach has previously been utilised in other analyses of the UK Biobank exome data, and extension of this work is beyond the scope of this paper. However future work should include the analyses of the exome data at a finer resolution to enable novel genetic discovery.

This study has offered the ability to study the genetics underlying complex retinal structures. Whilst OCT does not directly offer single-cell resolution, the quantitative values extracted from the images in the photoreceptor layer describe a single cell layer and capture variation in sub-cellular structures. It is interesting to note that some of the strong hits have gene expression in a different cell type. For example *RDH5* which is strongly associated with thickness of all three PRC layers is expressed in the RPE [57]. Therefore we are identifying genetic variation impacting sub-cellular morphology in a between cell type manner, highlighting the complex route between genetic variation, gene function and ultimate physiological differences. Imaging offers a methodology for interrogating such processes at a high-dimensional scale in a non-invasive manner and the protocol used here could be applied to other organs of the human body.

## Supporting information

S1 Fig Quantile-quantile plots of outer retinal thickness genome-wide association studies. (A) The quantile-quantile plot (qq-plot) for the GWAS of ONL thickness prior to meta-analysis (Lambda GC = 1.15, Intercept = 1.04, Ratio = 0.13). (B) The qq-plot for the GWAS of IS thickness prior to meta-analysis (Lambda GC = 1.07, Intercept = 1, Ratio <0). (C) The qq-plot for the GWAS of OS thickness prior to meta-analysis (Lambda GC = 1.09, Intercept = 1.02, Ratio = 0.15).
(PDF)

S2 Fig Exome-wide loss of function burden analysis study. Manhattan plot of p-values resulting from exome wide loss of function burden testing analysis for the thickness of each of the retinal layers (A) ONL, missense model, (B) IS, missense model, (C) OS, missense model, (D) ONL, loss of function model, (E) IS, loss of function model, (F) IS, loss of function model. Variants are considered significantly associated if they reach a a p-value threshold of P <5 × 10^-5^.
(PDF)

S3 Fig Comparison of the genetic effect on the thickness of concentric retinal fields. Plots depicting the residuals from models comparing the effect size of SNPs on the thickness of each retinal variants in different concentric retinal fields. Variants with significantly different effect sizes on the two different areas tested, as determined by a z-score, are highlighted in pink. (A) ONL, fovea compared to intermediate; (B) ONL, fovea compared to peripheral; (C) ONL, intermediate compared to peripheral; (D) IS, fovea compared to intermediate; (E) IS, fovea compared to peripheral; (F) IS, intermediate compared to peripheral; (G) OS, fovea compared to intermediate; (H) OS, fovea compared to peripheral; (I) OS, intermediate compared to peripheral.
(PDF)

S4 Fig Interaction between genetic variants in *VSX2* and *PRPH2*. Comparison of normalised outer segment (OS) thickness and outer nuclear layer thickness (ONL) in population subsetted by their genotype at *VSX2* (rs1972565) and *PRPH2* (rs375435).
(PDF)

S5 Fig Genetic discovery across analyses. A Venn diagram detailing the crossover in genetic discovery across the three different analysis types: genome-wide association study (GWAS), whole exome gene burden testing (Exome analysis) and the differential concentric genetic analysis (Differential concentric analysis). Genetic discovery refers to genes identified in exome analysis, or the associated gene to SNPs identified in GWAS and differential concentric analysis.
(PDF)

S1 Table Comparison of biological characteristics between whole UK Biobank population with OCT data and covariate data (n = 67,310), the group that passes our quality control criteria (n = 31,135), and the group that fails (n = 36,175). Results are presented as mean ± standard deviation. 2668 individuals, all within the failed filter group, have missing genetic sex data.
(PDF)

S2 Table Linkage score disequilibrium analysis results. Linkage score disequilibrium metrics for the three GWAS of the photoreceptor layers following analysis using LDSCore [20]. Metrics include lambda genomic control (GC), linkage disequilibrium score (LDSC) intercept and LDSC ratio.
(PDF)

S3 Table 111 SNPs significantly associated (P <5 × 10–8) with the thickness of one or more of the component photoreceptor cells (PRCs). Each SNP is annotated with the associated gene and any prior associations to ocular or non-ocular phenotypes. Variants considered to be representative of a single locus, examples of allelic heterogeneity, are highlighted in the same colour alternating white and blue. 27 novel SNPs, those that have no prior associations, are highlighted with an asterisk. Full results including effect size, effect allele specification and standard error are available in S4 Table.
(PDF)

S4 Table Expanded results of photoreceptor layer thickness genome-wide association studies. Extended results of GWAS of the three photoreceptor cell layers, the outer nuclear layer (ONL), inner segment (IS) and outer segment (OS) as well as the results selected by meta-analysis using MTAG, labelled MTAG. Each locus found significantly associated following meta-analysis is listed alongside the chromosome (Chr) and location (BP). The reference and alternative alleles are listed alongside the allele frequency (AF) of the alternative allele (A1). The effect size refers to the effect of having an additional copy of the A1 allele and the standard error (SE) is further listed.
(PDF)

S5 Table Selected rare monogenic ocular disease genes found to be associated with photoreceptor layer thicknesses in the present study. Several genes are involved in bringing about or shaping the photoreceptors’ electrical response to light (*RHO*, *GNAT2*, *SAG*, *GRK1*) or in recycling of the light-sensitive chromophore (*ABCA4*, *RLBP1*, *RBP3*, *RDH5*). Others are involved in maintaining outer retinal structure or in retinal and eye development. Superscript letters denote the relevant analyses in which the association was found: ^a^GWAS; ^b^differential association with ONL across concentric fields; ^c^differential association with IS across concentric fields; ^d^differential association with OS across concentric fields; ^e^exome analysis.
(PDF)

S6 Table List of genes associated with the thickness of the photoreceptor cell layers following exome analysis. List of genes associated with one of the three photoreceptor layers, the outer nuclear layer (ONL), inner segment (IS) and outer segment (OS) following gene burden testing. A significance threshold of P <5 × 10^-5^ is used. For each gene the gene name and chromosome is listed. Each gene is also annotated with any prior associations to ocular and non-ocular phenotypes. The number of individuals with the homozygous loss of function allele is listed as N. Two modes were tested, a missense (MS) model and a loss of function (LoF) model.
(PDF)

S7 Table Geneset enrichment analysis. The results of geneset enrichment analysis using DEPICT applied to the GWAS results of the meta analysed PRC layers. All genesets which were significantly enriched following correction for false discovery rate are listed below.
(PDF)

S8 Table Tissue enrichment analysis. The results of tissue enrichment analysis using DEPICT applied to the GWAS results of the meta analysed PRC layers. All Tissues which were nominally significantly enriched are listed below.
(PDF)

S9 Table List of SNPs differentially affecting the thickness of the ONL at the three concentric fields of the macula. List of SNPs with a significant z-score describing the differential effect on the ONL thickness at the foveal (F), intermediate (I) and peripheral (P) fields. The field (F1 or F2) and corresponding effect size from GWAS of thickness in each field are listed alongside the p-value of the comparative z-score. Each genetic variant is also annotated with associated gene and any ocular and non-ocular phenotypes previously associated with it. The different concentric comparisons are separated by bold horizontal lines.
(PDF)

S10 Table List of SNPs differentially affecting the thickness of the IS at the three concentric fields of the macula. List of SNPs with a significant z-score describing the differential effect on the IS thickness at the foveal (F), intermediate (I) and peripheral (P) fields. The field (F1 or F2) and corresponding effect size from GWAS of thickness in each field are listed alongside the p-value of the comparative z-score. Each genetic variant is also annotated with associated gene and any ocular and non-ocular phenotypes previously associated with it. The different concentric comparisons are separated by bold horizontal lines.
(PDF)

S11 Table List of SNPs differentially affecting the thickness of the OS at the three concentric fields of the macula. List of SNPs with a significant z-score describing the differential effect on the OS thickness at the foveal (F), intermediate (I) and peripheral (P) fields. The field (F1 or F2) and corresponding effect size from GWAS of thickness in each field are listed alongside the Bonferroni adjusted p-value of the comparative z-score. Each genetic variant is also annotated with associated gene and any ocular and non-ocular phenotypes previously associated it. The different concentric comparisons are separated by bold horizontal lines.
(PDF)

S1 Text A list of the members of the UK Biobank Eye and Vision Consortium. (PDF)

## References

[1] Tick S, Rossant F, Ghorbel I, Gaudric A, Sahel JA, Chaumet-Riffaud P, et al. Foveal Shape and Structure in a Normal Population. Investigative Ophthalmology & Visual Science. 2011;52:5105–5110. doi: 10.1167/iovs.10-7005 21803966

[2] Chopra R, Wagner SK, Keane PA. Optical coherence tomography in the 2020s—outside the eye clinic. Eye 2020 35:1. 2020;35:236–243. doi: 10.1038/s41433-020-01263-6 33168975 PMC7853067

[3] Fritsche LG, Igl W, Bailey JNC, Grassmann F, Sengupta S, Bragg-Gresham JL, et al. A large genome-wide association study of age-related macular degeneration highlights contributions of rare and common variants. Nature Genetics. 2016;48:134–143. doi: 10.1038/ng.3448 26691988 PMC4745342

[4] Chakravarthy U, Evans J, Rosenfeld PJ. Age related macular degeneration. BMJ. 2010;340:526–530. doi: 10.1136/bmj.c981 20189972

[5] Pontikos N, Arno G, Jurkute N, Schiff E, Ba-Abbad R, Malka S, et al. Genetic Basis of Inherited Retinal Disease in a Molecularly Characterized Cohort of More Than 3000 Families from the United Kingdom. Ophthalmology. 2020;127:1384–1394. doi: 10.1016/j.ophtha.2020.04.008 32423767 PMC7520514

[6] Khawaja AP, Chua S, Hysi PG, Georgoulas S, Currant H, Fitzgerald TW, et al. Comparison of Associations with Different Macular Inner Retinal Thickness Parameters in a Large Cohort: The UK Biobank. Ophthalmology. 2020;127. doi: 10.1016/j.ophtha.2019.08.015 31585827

[7] Pilat AV, Proudlock FA, Mohammad S, Gottlob I. Normal macular structure measured with optical coherence tomography across ethnicity. British Journal of Ophthalmology. 2014;98:941–945. doi: 10.1136/bjophthalmol-2013-303119 24518076

[8] Gao XR, Huang H, Kim H. Genome-wide association analyses identify 139 loci associated with macular thickness in the UK Biobank cohort. Human Molecular Genetics. 2019;28:1162–1172. doi: 10.1093/hmg/ddy422 30535121 PMC6423416

[9] Currant H, Hysi P, Fitzgerald TW, Gharahkhani P, Bonnemaijer PWM, Senabouth A, et al. Genetic variation affects morphological retinal phenotypes extracted from UK Biobank optical coherence tomography images. PLOS Genetics. 2021;17:e1009497. doi: 10.1371/journal.pgen.1009497 33979322 PMC8143408

[10] Elliott P, on behalf of UK Biobank, Peakman TC, on behalf of UK Biobank. The UK Biobank sample handling and storage protocol for the collection, processing and archiving of human blood and urine. International Journal of Epidemiology. 2008;37:234–244. doi: 10.1093/ije/dym276 18381398

[11] Cumberland PM, Rahi JS, for the UK Biobank Eye, Consortium V. Visual Function, Social Position, and Health and Life Chances: The UK Biobank Study. JAMA Ophthalmology. 2016;134:959–966. 27466983 10.1001/jamaophthalmol.2016.1778

[12] Keane PA, Grossi CM, Foster PJ, Yang Q, Reisman CA, Chan K, et al. Optical Coherence Tomography in the UK Biobank Study—Rapid Automated Analysis of Retinal Thickness for Large Population-Based Studies. PLOS ONE. 2016;11:e0164095. doi: 10.1371/journal.pone.0164095 27716837 PMC5055325

[13] Khawaja AP, Bailey JNC, Wareham NJ, Scott RA, Simcoe M, Igo RP, et al. Genome-wide analyses identify 68 new loci associated with intraocular pressure and improve risk prediction for primary open-angle glaucoma. Nature Genetics 2018 50:6. 2018;50:778–782. doi: 10.1038/s41588-018-0126-8 29785010 PMC5985943

[14] Yang Q, Reisman CA, Wang Z, Fukuma Y, Hangai M, Yoshimura N, et al. Automated layer segmentation of macular OCT images using dual-scale gradient information. Optics express. 2010;18:21293. doi: 10.1364/OE.18.021293 20941025 PMC3101081

[15] Camparini M, Cassinari P, Ferrigno L, Macaluso C. ETDRS-fast: implementing psychophysical adaptive methods to standardized visual acuity measurement with ETDRS charts. Investigative Ophthalmology & Visual Science. 2001;42:1226–1231. 11328731

[16] Chang CC, Chow CC, Tellier LCAM, Vattikuti S, Purcell SM, Lee JJ. Second-generation PLINK: Rising to the challenge of larger and richer datasets. GigaScience. 2015;4:7. doi: 10.1186/s13742-015-0047-8 25722852 PMC4342193

[17] Altshuler DM, Gibbs RA, Peltonen L, Schaffner SF, Yu F, Dermitzakis E, et al. Integrating common and rare genetic variation in diverse human populations. Nature 2010 467:7311. 2010;467:52–58. doi: 10.1038/nature09298 20811451 PMC3173859

[18] Bycroft C, Freeman C, Petkova D, Band G, Elliott LT, Sharp K, et al. The UK Biobank resource with deep phenotyping and genomic data. Nature. 2018;562:203–209. doi: 10.1038/s41586-018-0579-z 30305743 PMC6786975

[19] Patel PJ, Foster PJ, Grossi CM, Keane PA, Ko F, Lotery A, et al. Spectral-Domain Optical Coherence Tomography Imaging in 67 321 Adults: Associations with Macular Thickness in the UK Biobank Study. Ophthalmology. 2016;123:829–840. doi: 10.1016/j.ophtha.2015.11.009 26746598

[20] Bulik-Sullivan BK, Loh PR, Finucane HK, Ripke S, Yang J, Patterson N, et al. LD Score regression distinguishes confounding from polygenicity in genome-wide association studies. Nature Genetics 2015 47:3. 2015;47:291–295. doi: 10.1038/ng.3211 25642630 PMC4495769

[21] Turley P, Walters RK, Maghzian O, Okbay A, Lee JJ, Fontana MA, et al. Multi-trait analysis of genome-wide association summary statistics using MTAG. Nature Genetics. 2018;50:229–237. doi: 10.1038/s41588-017-0009-4 29292387 PMC5805593

[22] Yang J, Ferreira T, Morris AP, Medland SE, Madden PAF, Heath AC, et al. Conditional and joint multiple-SNP analysis of GWAS summary statistics identifies additional variants influencing complex traits. Nature Genetics. 2012;44:369–375. doi: 10.1038/ng.2213 22426310 PMC3593158

[23] Pruim RJ, Welch RP, Sanna S, Teslovich TM, Chines PS, Gliedt TP, et al. LocusZoom: regional visualization of genome-wide association scan results. Bioinformatics (Oxford, England). 2010;26:2336–2337. doi: 10.1093/bioinformatics/btq419 20634204 PMC2935401

[24] McLaren W, Gil L, Hunt SE, Riat HS, Ritchie GRS, Thormann A, et al. The Ensembl Variant Effect Predictor. Genome Biology. 2016;17:122. doi: 10.1186/s13059-016-0974-4 27268795 PMC4893825

[25] Howe KL, Achuthan P, Allen J, Allen J, Alvarez-Jarreta J, Amode MR, et al. Ensembl 2021. Nucleic Acids Research. 2021;49:D884–D891. doi: 10.1093/nar/gkaa942 33137190 PMC7778975

[26] Ghoussaini M, Mountjoy E, Carmona M, Peat G, Schmidt EM, Hercules A, et al. Open Targets Genetics: Systematic identification of trait-associated genes using large-scale genetics and functional genomics. Nucleic Acids Research. 2021;49:D1311–D1320. doi: 10.1093/nar/gkaa840 33045747 PMC7778936

[27] Pers TH, Karjalainen JM, Chan Y, Westra HJ, Wood AR, Yang J, et al. Biological interpretation of genome-wide association studies using predicted gene functions. Nature Communications. 2015;6:1–9. doi: 10.1038/ncomms6890 25597830 PMC4420238

[28] Reis LM, Khan A, Kariminejad A, Ebadi F, Tyler RC, Semina EV. VSX2 mutations in autosomal recessive microphthalmia. Molecular Vision. 2011;17:2527–2532. 21976963 PMC3185030

[29] Gonzalez-Fernandez F, Kurz D, Bao Y, Newman S, Conway BP, Young JE, et al. 11-cis Retinol dehydrogenase mutations as a major cause of the congenital night-blindness disorder known as fundus albipunctatus. Molecular Vision. 1999;5:41–41. 10617778

[30] Rotenstreich Y, Harats D, Shaish A, Pras E, Belkin M. Treatment of a retinal dystrophy, fundus albipunctatus, with oral 9-cis–carotene. British Journal of Ophthalmology. 2010;94:616–621. doi: 10.1136/bjo.2009.167049 19955196

[31] Saari JC, Nawrot M, Kennedy BN, Garwin GG, Hurley JB, Huang J, et al. Visual cycle impairment in cellular retinaldehyde binding protein (CRALBP) knockout mice results in delayed dark adaptation. Neuron. 2001;29:739–748. doi: 10.1016/S0896-6273(01)00248-3 11301032

[32] Wu Z, Hasan A, Liu T, Teller DC, Crabb JW. Identification of CRALBP ligand interactions by photoaffinity labeling, hydrogen/deuterium exchange, and structural modeling. Journal of Biological Chemistry. 2004;279:27357–27364. doi: 10.1074/jbc.M401960200 15100222

[33] Hipp S, Zobor G, Glöckle N, Mohr J, Kohl S, Zrenner E, et al. Phenotype variations of retinal dystrophies caused by mutations in the RLBP1 gene. Acta Ophthalmologica. 2015;93:e281–e286. doi: 10.1111/aos.12573 25429852

[34] den Hollander AI, McGee TL, Ziviello C, Banfi S, Dryja TP, Gonzalez-Fernandez F, et al. A homozygous missense mutation in the IRBP gene (RBP3) associated with autosomal recessive retinitis pigmentosa. Investigative Ophthalmology and Visual Science. 2009;50:1864–1872. doi: 10.1167/iovs.08-2497 19074801 PMC2823395

[35] Arno G, Hull S, Robson AG, Holder GE, Cheetham ME, Webster AR, et al. Lack of Interphotoreceptor Retinoid Binding Protein Caused by Homozygous Mutation of RBP3 Is Associated With High Myopia and Retinal Dystrophy. Investigative Ophthalmology & Visual Science. 2015;56:2358–2365. doi: 10.1167/iovs.15-16520 25766589

[36] Xie Y, Gonome T, Yamauchi K, Maeda-Monai N, Tanabu R, ichi Ishiguro S, et al. A spectral-domain optical coherence tomographic analysis of Rdh5-/- mice retina. PLOS ONE. 2020;15:e0231220. doi: 10.1371/journal.pone.0231220 32271812 PMC7144952

[37] Hayashi T, Tsuzuranuki S, Kozaki K, Urashima M, Tsuneoka H. Macular dysfunction in Oguchi disease with the frequent mutation 1147delA in the SAG gene. Ophthalmic Research. 2011;46:175–180. doi: 10.1159/000325024 21447990

[38] Hashimoto H, Kishi S. Shortening of the rod outer segment in Oguchi disease. Graefe’s Archive for Clinical and Experimental Ophthalmology. 2009;247:1561–1563. doi: 10.1007/s00417-009-1114-6 19513740

[39] Plotnikov D, Shah RL, Rodrigues JN, Cumberland PM, Rahi JS, Hysi PG, et al. A commonly occurring genetic variant within the NPLOC4-TSPAN10-PDE6G gene cluster is associated with the risk of strabismus. Human Genetics 2019 138:7. 2019;138:723–737. doi: 10.1007/s00439-019-02022-8 31073882 PMC6611893

[40] Kaye RA, Patasova K, Patel PJ, Hysi P, Lotery AJ, Patel PJ, et al. Macular thickness varies with age-related macular degeneration genetic risk variants in the UK Biobank cohort. Scientific Reports 2021 11:1. 2021;11:1–11. doi: 10.1038/s41598-021-02631-2 34853365 PMC8636487

[41] Zekavat SM, Sekimitsu S, Ye Y, Raghu V, Zhao H, Elze T, et al. Photoreceptor layer thinning is an early biomarker for age-related macular degeneration: Epidemiological and genetic evidence from UK Biobank optical coherence tomography data. Ophthalmology. 2022. doi: 10.1016/j.ophtha.2022.02.001 PMC9134644 35149155

[42] Stone EM, Andorf JL, Whitmore SS, DeLuca AP, Giacalone JC, Streb LM, et al. Clinically Focused Molecular Investigation of 1000 Consecutive Families with Inherited Retinal Disease. Ophthalmology. 2017;124:1314–1331. doi: 10.1016/j.ophtha.2017.04.008 28559085 PMC5565704

[43] Cheng L, Yu H, Jiang Y, He J, Pu S, Li X, et al. Identification of a novel MYO7A mutation in Usher syndrome type 1. Oncotarget. 2018;9:2295. doi: 10.18632/oncotarget.23408 29416772 PMC5788640

[44] Peng GH, Ahmad O, Ahmad F, Liu J, Chen S. The photoreceptor-specific nuclear receptor Nr2e3 interacts with Crx and exerts opposing effects on the transcription of rod versus cone genes. Human molecular genetics. 2005;14:747–764. doi: 10.1093/hmg/ddi070 15689355

[45] Li S, Datta S, Brabbit E, Love Z, Woytowicz V, Flattery K, et al. Nr2e3 is a genetic modifier that rescues retinal degeneration and promotes homeostasis in multiple models of retinitis pigmentosa. Gene Therapy 2020 28:5. 2020;28:223–241. doi: 10.1038/s41434-020-0134-z 32123325 PMC7483267

[46] Kent OA, Saha M, Coyaud E, Burston HE, Law N, Dadson K, et al. Haploinsufficiency of RREB1 causes a Noonan-like RASopathy via epigenetic reprogramming of RAS-MAPK pathway genes. Nature Communications 2020 11:1. 2020;11:1–12. doi: 10.1038/s41467-020-18483-9 32938917 PMC7495420

[47] Harding SR, Bocchetta M, Gordon E, Cash DM, Cardoso MJ, Druyeh R, et al. The TMEM106B risk allele is associated with lower cortical volumes in a clinically diagnosed frontotemporal dementia cohort. Journal of Neurology, Neurosurgery & Psychiatry. 2017;88:997–998. doi: 10.1136/jnnp-2017-315641 28446602 PMC5740537

[48] Kreeke JAVD, Nguyen HT, Konijnenberg E, Tomassen J, Braber AD, Kate MT, et al. Optical coherence tomography angiography in preclinical Alzheimer’s disease. British Journal of Ophthalmology. 2020;104:157–161. doi: 10.1136/bjophthalmol-2019-314127 31118186 PMC7025728

[49] Chan VTT, Sun Z, Tang S, Chen LJ, Wong A, Tham CC, et al. Spectral-Domain OCT Measurements in Alzheimer’s Disease: A Systematic Review and Meta-analysis. Ophthalmology. 2019;126:497–510. doi: 10.1016/j.ophtha.2018.08.009 30114417 PMC6424641

[50] Evans DR, Green JS, Johnson GJ, Schwartzentruber J, Majewski J, Beaulieu CL, et al. Novel 25 kb Deletion of MERTK Causes Retinitis Pigmentosa With Severe Progression. Investigative Ophthalmology & Visual Science. 2017;58:1736–1742. doi: 10.1167/iovs.16-20864 28324114

[51] Stingl K, Mayer AK, Llavona P, Mulahasanovic L, Rudolph G, Jacobson SG, et al. CDHR1 mutations in retinal dystrophies. Scientific Reports 2017 7:1. 2017;7:1–11. doi: 10.1038/s41598-017-07117-8 28765526 PMC5539332

[52] de Sompele SV, Smith C, Karali M, Corton M, Schil KV, Peelman F, et al. Biallelic sequence and structural variants in RAX2 are a novel cause for autosomal recessive inherited retinal disease. Genetics in Medicine 2018 21:6. 2018;21:1319–1329. doi: 10.1038/s41436-018-0345-5 30377383 PMC6752271

[53] Conley SM, Naash MI. Gene Therapy for PRPH2-Associated Ocular Disease: Challenges and Prospects. Cold Spring Harbor Perspectives in Medicine. 2014;4. doi: 10.1101/cshperspect.a017376 25167981 PMC4208711

[54] Boon CJF, den Hollander AI, Hoyng CB, Cremers FPM, Klevering BJ, Keunen JEE. The spectrum of retinal dystrophies caused by mutations in the peripherin/RDS gene. Progress in Retinal and Eye Research. 2008;27:213–235. doi: 10.1016/j.preteyeres.2008.01.002 18328765

[55] Michaelides M, Holder GE, Bradshaw K, Hunt DM, Moore AT. Cone-rod dystrophy, intrafamilial variability, and incomplete penetrance associated with the R172W mutation in the peripherin/RDS gene. Ophthalmology. 2005;112:1592–1598. doi: 10.1016/j.ophtha.2005.04.004 16019073

[56] Neuhofer CM, Catarino CB, Schmidt H, Seelos K, Alhaddad B, Haack TB, et al. LINS1-associated neurodevelopmental disorder: Family with novel mutation expands the phenotypic spectrum. Neurology: Genetics. 2020;6:500. doi: 10.1212/NXG.0000000000000500 32802957 PMC7413627

[57] Wen B, Li S, Li H, Chen Y, Ma X, Wang J, et al. Microphthalmia-associated transcription factor regulates the visual cycle genes Rlbp1 and Rdh5 in the retinal pigment epithelium. Scientific Reports 2016 6:1. 2016;6:1–9. doi: 10.1038/srep21208 26876013 PMC4753414

