# Supplementary figures and images for: Sub-cellular level resolution of common genetic variation in the photoreceptor layer identifies continuum between rare disease and common variation
Source: PLoS Genet. 2023 Feb 27;19(2):e1010587. doi: 10.1371/journal.pgen.1010587 (PMC9997913; doi:10.1371/journal.pgen.1010587)

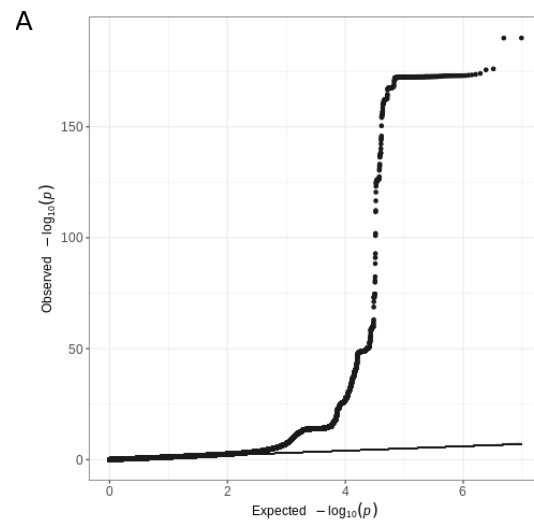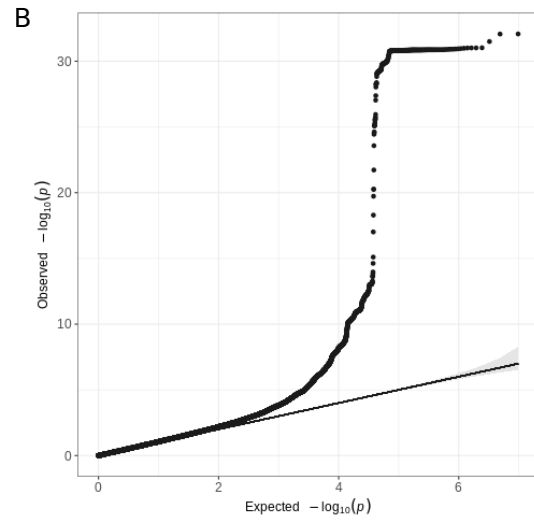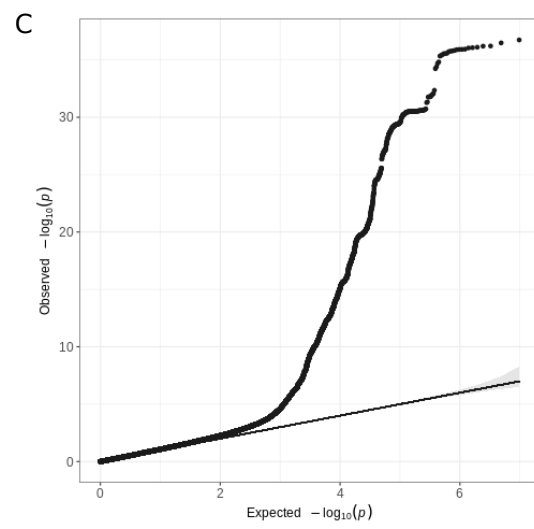

Supplement: S1 Fig — (A) The quantile-quantile plot (qq-plot) for the GWAS of ONL thickness prior to meta-analysis (Lambda GC = 1.15, Intercept = 1.04, Ratio = 0.13). (B) The qq-plot for the GWAS of IS thickness prior to meta-analysis (Lambda GC = 1.07, Intercept = 1, Ratio <0). (C) The qq-plot for the GWAS of OS thickness prior to meta-analysis (Lambda GC = 1.09, Intercept = 1.02, Ratio = 0.15). (PDF) [file pgen.1010587.s001.pdf]

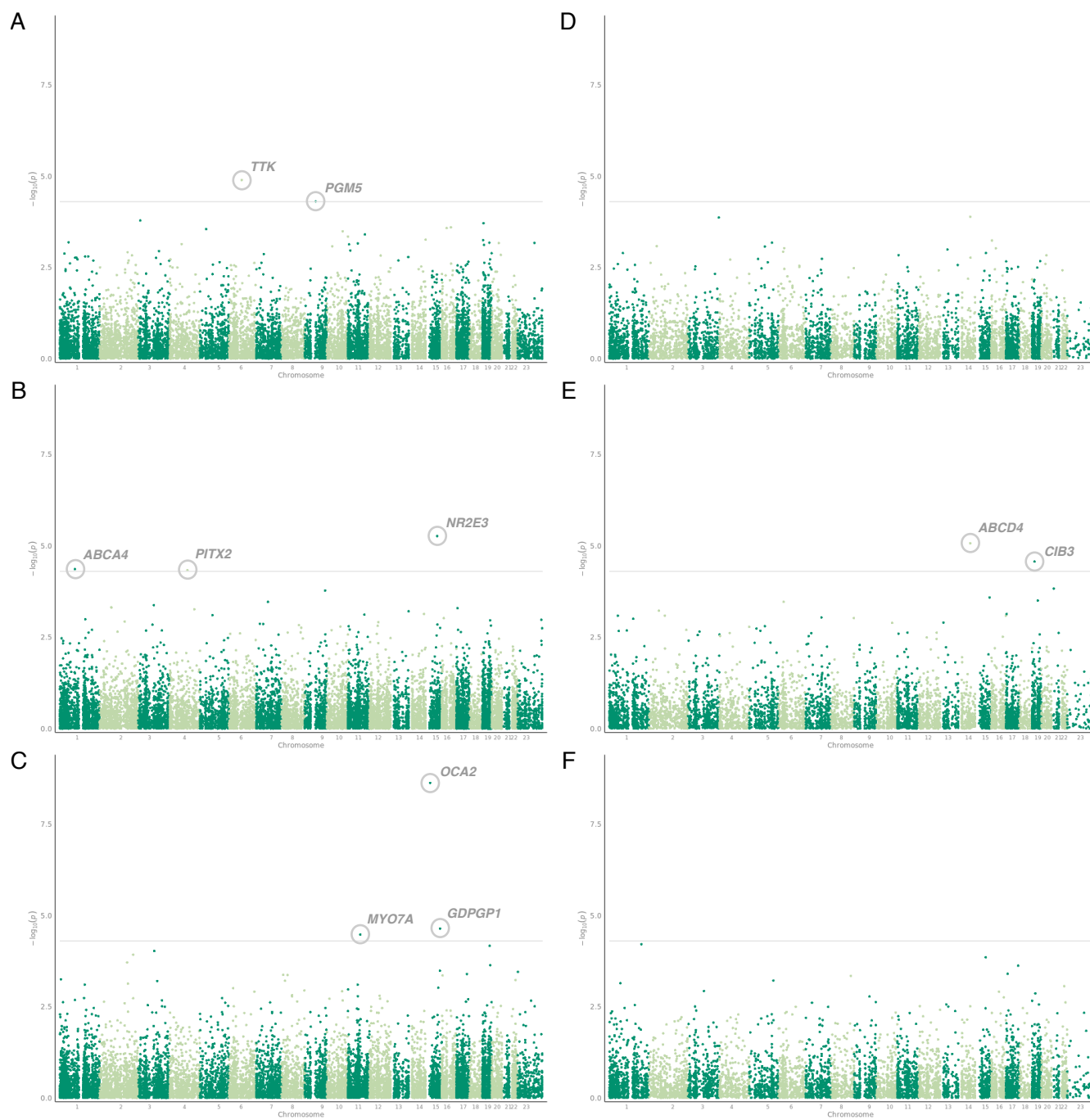

Supplement: S2 Fig — Manhattan plot of p-values resulting from exome wide loss of function burden testing analysis for the thickness of each of the retinal layers (A) ONL, missense model, (B) IS, missense model, (C) OS, missense model, (D) ONL, loss of function model, (E) IS, loss of function model, (F) IS, loss of function model. Variants are considered significantly associated if they reach a a p-value threshold of P <5 × 10^-5^. (PDF) [file pgen.1010587.s002.pdf]

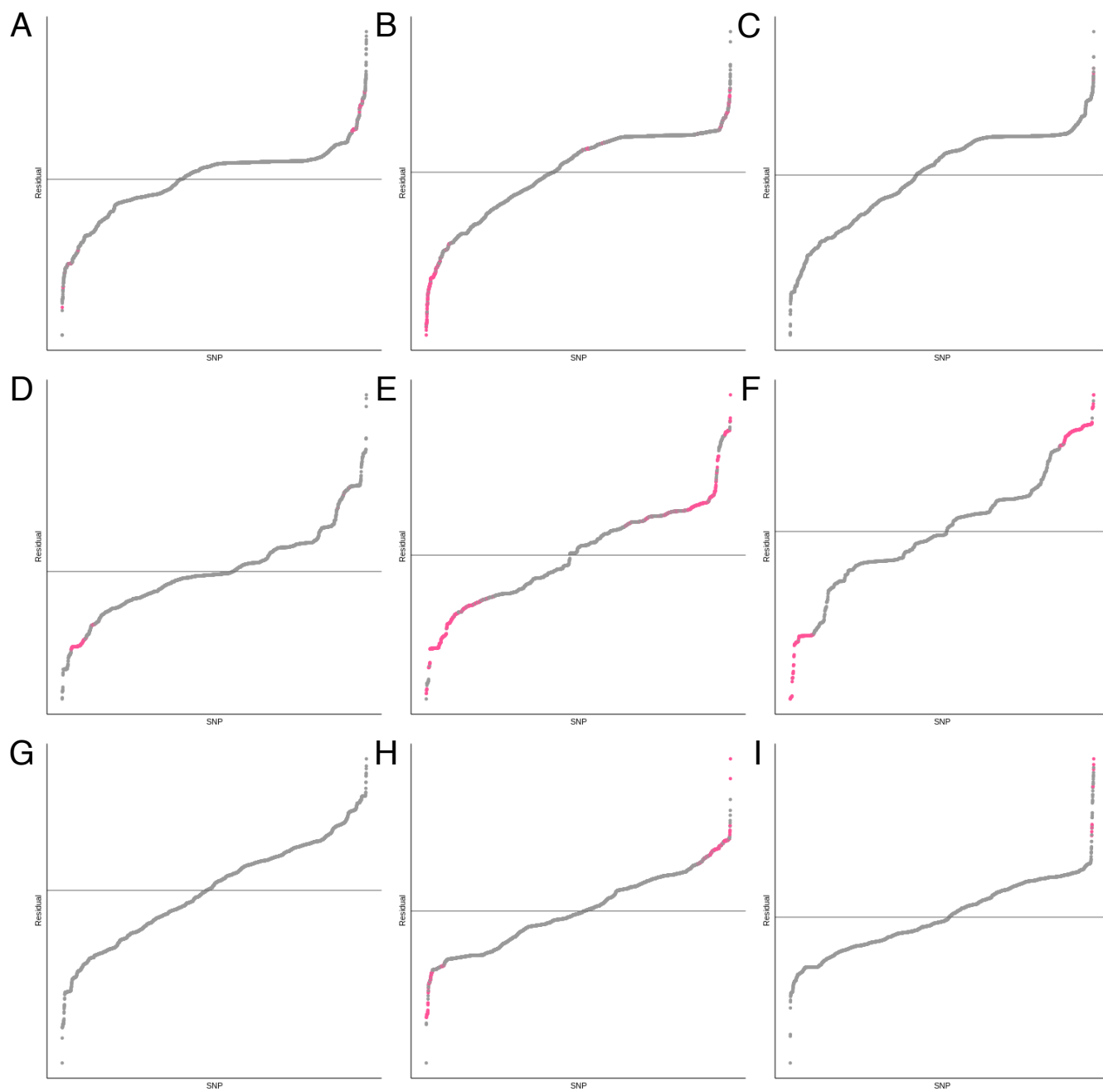

Supplement: S3 Fig — Plots depicting the residuals from models comparing the effect size of SNPs on the thickness of each retinal variants in different concentric retinal fields. Variants with significantly different effect sizes on the two different areas tested, as determined by a z-score, are highlighted in pink. (A) ONL, fovea compared to intermediate; (B) ONL, fovea compared to peripheral; (C) ONL, intermediate compared to peripheral; (D) IS, fovea compared to intermediate; (E) IS, fovea compared to peripheral; (F) IS, intermediate compared to peripheral; (G) OS, fovea compared to intermediate; (H) OS, fovea compared to peripheral; (I) OS, intermediate compared to peripheral. (PDF) [file pgen.1010587.s003.pdf]

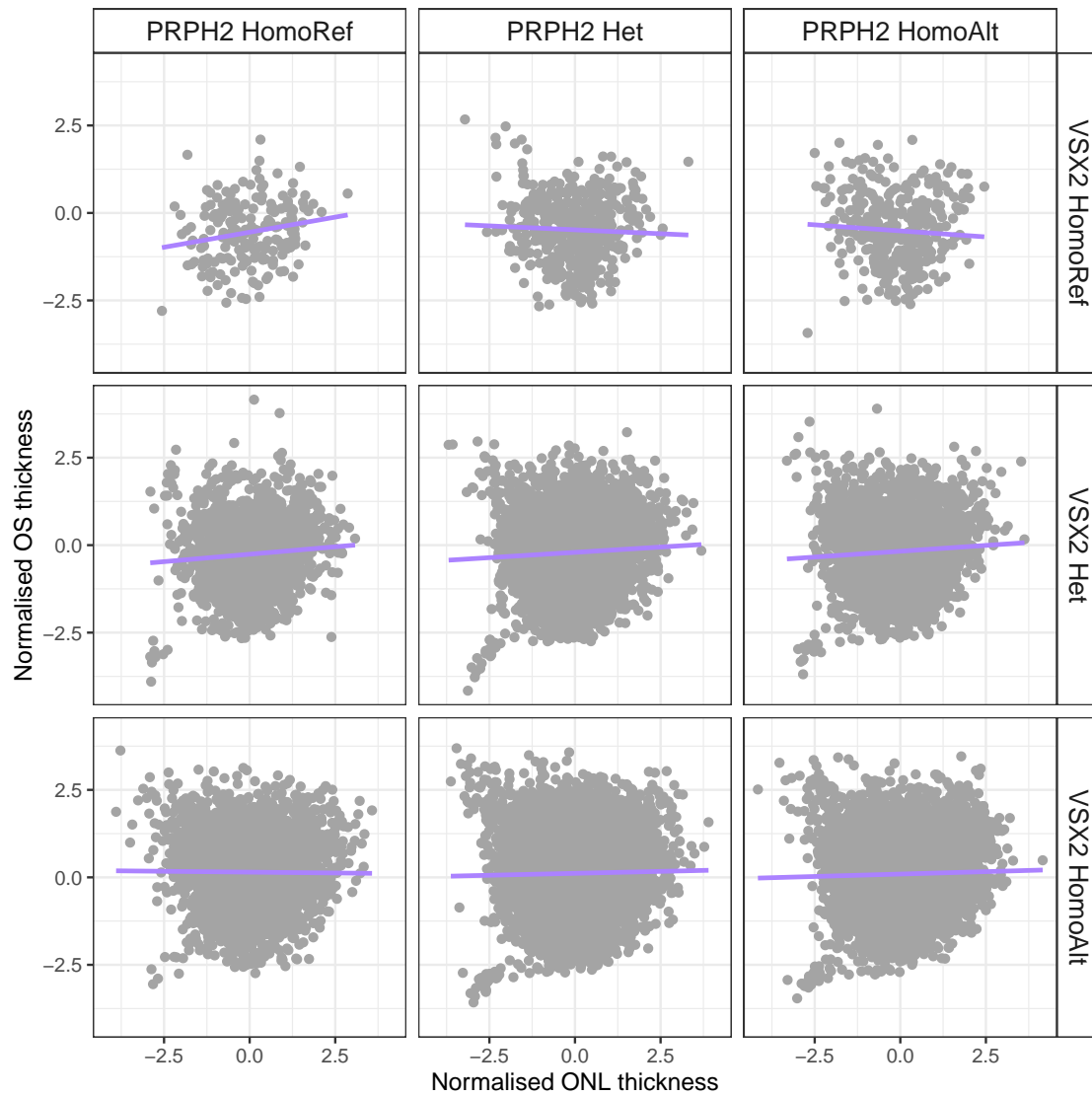

Supplement: S4 Fig — Comparison of normalised outer segment (OS) thickness and outer nuclear layer thickness (ONL) in population subsetted by their genotype at VSX2 (rs1972565) and PRPH2 (rs375435). (PDF) [file pgen.1010587.s004.pdf]

GWAS

Exome analysis

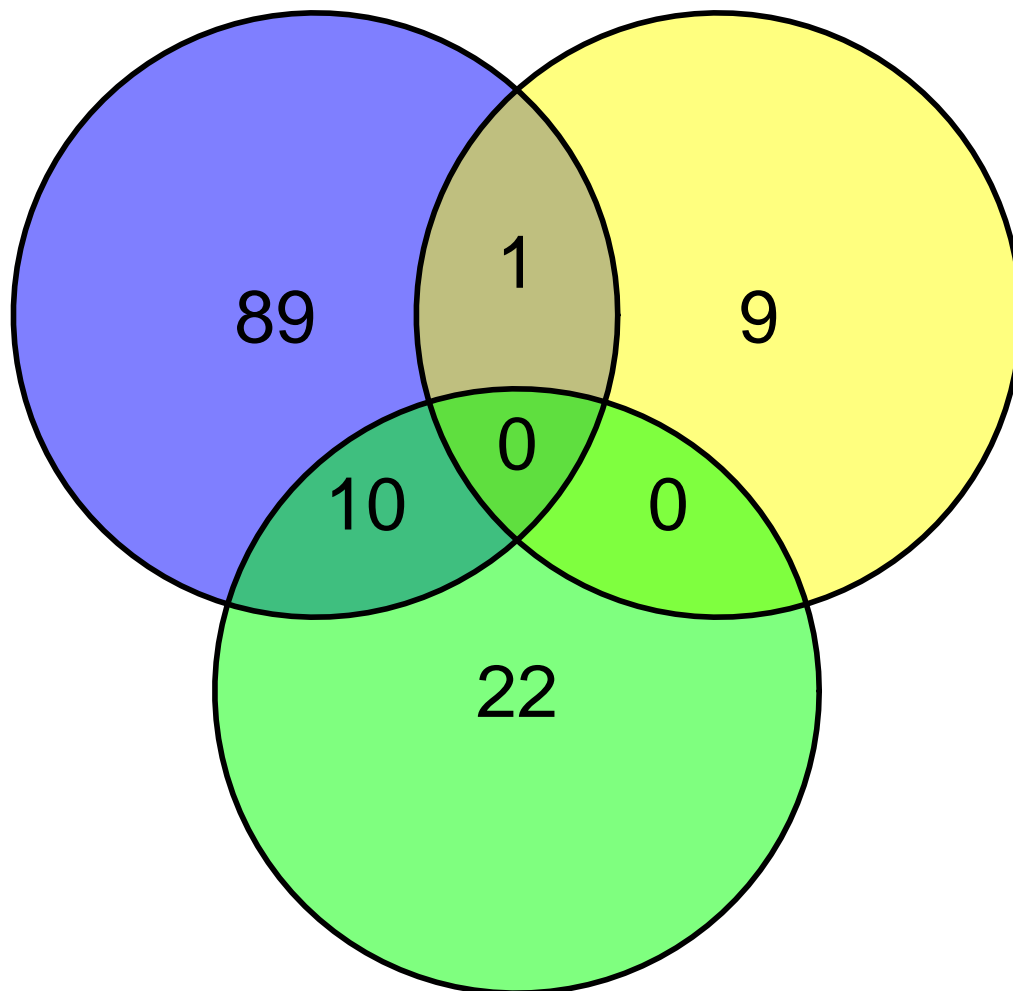

Differential concentric analysis

Supplement: S5 Fig — A Venn diagram detailing the crossover in genetic discovery across the three different analysis types: genome-wide association study (GWAS), whole exome gene burden testing (Exome analysis) and the differential concentric genetic analysis (Differential concentric analysis). Genetic discovery refers to genes identified in exome analysis, or the associated gene to SNPs identified in GWAS and differential concentric analysis. (PDF) [file pgen.1010587.s005.pdf]
