# Supplementary material for: Sub-cellular level resolution of common genetic variation in the photoreceptor layer identifies continuum between rare disease and common variation
Source: PLoS Genet. 2023 Feb 27;19(2):e1010587. doi: 10.1371/journal.pgen.1010587 (PMC9997913; doi:10.1371/journal.pgen.1010587)
Supplement: S1 Table — Results are presented as mean ± standard deviation. 2668 individuals, all within the failed filter group, have missing genetic sex data. (PDF) [file pgen.1010587.s006.pdf]

	**Total OCT Population**	**Pass filter**	**Fail filter**
Height (cm)	168.68 ± 9.25	169.43 ± 9.15	168.03 ± 9.29
Age (years)	57 ± 8	57 ± 8	57 ± 8
Weight (Kg)	78.12 ± 16.02	78.47 ± 15.82	77.82 ± 16.17
Sex (m/f)	29,713/34,929	14,696/16,439	15,017/18,490
Refractive Error Left (Dioptres)	-0.32 ± 2.73	0.23 ± 1.49	-0.80 ± 3.41
Refractive Error Right (Dioptres)	-0.38±2.73	0.18 ± 1.48	-0.87 ± 3.40
ONL thickness	79.79 ±9.03	80.75 ± 6.44	78.89 ± 10.84
IS thickness	24.10 ± 2.97	23.60 ± 1.67	24.58 ± 3.75
OS thickness	37.22 ± 5.65	38.04 ± 3.90	36.44 ± 6.81
