# Supplementary material for: Sub-cellular level resolution of common genetic variation in the photoreceptor layer identifies continuum between rare disease and common variation
Source: PLoS Genet. 2023 Feb 27;19(2):e1010587. doi: 10.1371/journal.pgen.1010587 (PMC9997913; doi:10.1371/journal.pgen.1010587)
Supplement: S2 Table — Linkage score disequilibrium metrics for the three GWAS of the photoreceptor layers following analysis using LDSCore [20]. Metrics include lambda genomic control (GC), linkage disequilibrium score (LDSC) intercept and LDSC ratio. (PDF) [file pgen.1010587.s007.pdf]

GWAS phenotype	lambda GC	LDSC intercept	LDSC ratio
ONL	1.15	1.04	0.13
IS	1.07	1	<0
OS	1.09	1.02	0.15
