# Supplementary material for: Sub-cellular level resolution of common genetic variation in the photoreceptor layer identifies continuum between rare disease and common variation
Source: PLoS Genet. 2023 Feb 27;19(2):e1010587. doi: 10.1371/journal.pgen.1010587 (PMC9997913; doi:10.1371/journal.pgen.1010587)
Supplement: S3 Table — Each SNP is annotated with the associated gene and any prior associations to ocular or non-ocular phenotypes. Variants considered to be representative of a single locus, examples of allelic heterogeneity, are highlighted in the same colour alternating white and blue. 27 novel SNPs, those that have no prior associations, are highlighted with an asterisk. Full results including effect size, effect allele specification and standard error are available in S4 Table. (PDF) [file pgen.1010587.s008.pdf]

SNP	Chr	P-value	Associated Gene	Ocular Phenotypes	Non-ocular Phenotypes
rs112248193	1	2.73E-09	*LRRC47*		High light scatter reticulocyte count
rs2128416	1	4.83E-13	*CASZ1*		Amlodipine, High blood pressure, Hypertension
rs11587687	1	5.02E-09	*GNAT2*		Frequency of tiredness/lethargy
rs3790612	1	2.60E-15	*ST7L*		Blood pressure, High blood pressure, Platelet distribution width
rs72683442	1	4.13E-21	*SLC16A1*	Spherical power	Birth weight
rs410895	1	8.19E-22	*CFHR2*	Macular degeneration	
rs6427827	1	2.50E-16	*ZNF281*	Age started wearing glasses, Spherical power	Urinary albumin excretion
rs11577827 *	1	2.77E-13	*KDM5B*		
rs919655	1	2.36E-09	*PROX1-AS1*		Mean sphered cell volume, Oily fish intake
rs12140498	1	3.14E-09	*LINC02257*		Arm fat percentage, Colon cancer
rs6426584	1	7.03E-11	*CDC42BPA*		Impedance of arms, Neutrophil traits
rs7594221	2	1.07E-14	*ATAD2B*		Forced vital capacity, Height, Intelligence, Lymphocyte volume, Mean sphered cell volume, Platelet traits, Reticulocyte traits
rs116350483	2	2.10E-26	*LINC01412*		Particulate matter air pollution
rs80265589	2	1.86E-13	*STK39*		Impedance of arm
rs28416292 *	2	1.78E-08	*CERKL*		
rs58172089	2	2.74E-08	*PLCL1*		Arm fat, Body mass index, Impedance of body
rs3755152 *	2	4.39E-10	*MREG*		
rs148388367 *	2	1.20E-21	*MREG*		
rs201030469	2	6.58E-09	*TNS1*		Breast cancer

rs7564805	2	6.16E-29	*SAG*		Crohn's disease, Inflammatory Bowel Disease
rs34234056 *	3	2.37E-08	*LINC01267*		
rs11129176	3	1.53E-08	*RARB*	Optic disc area	Height, Red blood cell count
rs6775323 *	3	5.79E-15	*LINC02084*		
rs62282867	3	4.63E-19	*IMPG2*		Heel bone mineral density
rs111163508 *	3	2.28E-20	*RHO*		
rs7430585	3	1.26E-10	*TSC22D2*	Age started wearing glasses	
rs115237855 *	5	3.73E-12	*BASP1-AS1*		
rs78303234 *	5	3.32E-08	*BASP1*		
rs30373	5	7.70E-10	*LINC01948*	Macular thickness	
rs63338061	5	9.81E-11	*MAP1B*	Age started wearing glasses	
rs17421627	5	3.30E-33	*LINC00461*	Macular thickness, Retinal vascular calliber	Comparative height at age 10, Seen doctor for nerves, anxiety, tension or depression
rs62391700	5	5.05E-13	*LMNB1-DT*		Height, Multiple myeloma
rs1109114	5	2.65E-13	*AFAP1L1*		Body mass index, Forced expiratory volume, High blood pressure, Hearing difficulties, Hypertension
rs1438692	5	1.32E-11	*AFAP1L1*		Forced expiratory volume
rs6875105	5	1.39E-13	*BOD1*	Refractive error	
rs2326838	6	7.86E-13	*RREB1*	Age started wearing glasses, Spherical equivalent, Spherical power	Lymphocyte traits, White blood cell count
rs12192672	6	8.88E-10	*RREB1*		Haemoglobin traits, Hair/balding pattern, Height, Red blood cell traits
rs17507554	6	3.93E-09	*NEDD9*	Macular thickness	

rs6923949	6	4.22E-09	*TULP1*		Ankle spacing width, Anthropometric traits, Basal metabolic rates, Forced expiratory volume, White blood cell count
rs375435	6	3.56E-16	*PRPH2*		Neutrophil percentage
rs6910414	6	1.87E-10	*DST-AS1*		Haemoglobin concentration, Red blood cell count
rs947340 *	6	1.17E-13	*IMPG1*		
rs74526772	6	1.13E-19	*ATG5*		Asthma
rs9639276	7	4.74E-10	*SUN1*		Body mass index, Forced expiratory volume, Impedance of body
rs12531825	7	5.94E-09	*GLCCI1-DT*		Average total household income, Educational attainment, Forced expiratory volume, Height, Leg fat, Time spent watching TV
rs12719025	7	6.39E-22	*COBL*	Macular thickness, Refractive error, Spherical power, Strong/weak meridian	
rs111963714	7	5.96E-13	*PILRB*		Platelet traits
rs34926272 *	7	2.03E-11	*UBE2H*		
rs62490856	8	5.02E-13	*RP1L1*	Ocult macular dystrophy, Strong meridian	Body mass index, Forced expiratory volume, Heel bone mineral density, Myeloid white blood cell count, Platelet distribution width, Red blood cell distribution width
rs61675430	8	1.30E-10	*CHD7*		Lymphocyte traits, Neutrophil traits, Potassium in urine
rs13263941	8	2.07E-50	*RSPO2*	Strong/weak meridian	Coffee intake, Hair colour
rs376067714	8	2.73E-24	*RSPO2*		
rs9298817	9	2.90E-22	*MIR31HG*	Strong meridian	Hypothyroidism

rs10781177	9	1.49E-08	*RORB*	Age started wearing glasses, Spherical power	
rs717299	9	3.80E-13	*RORB*		Impedance of arm
rs111245635	10	8.41E-10	*RBP3*	Age started wearing glasses, Retinitis pigmentosa, Strong/weak meridian	Soya milk
rs1947075	10	7.86E-10	*ARHGAP22*	Macular thickness	
rs7916697	10	3.80E-13	*ATOH7*	Cup-disc ratio, Optic cup area, Optic disc area, Optic disc radius	Anthropometric traits, Basal metabolic rate, Forced expiratory volume, Gastro-oesophageal reflux
rs11200922	10	1.62E-26	*CDHR1*	Age started wearing glasses, Hypermetropia, Spherical power	
rs34309160 *	10	3.34E-08	*GBF1*		
rs17102399	10	2.44E-08	*FGFR2*		Breast cancer
rs60401382	10	2.43E-16	*HTRA1*	Macular degeneration, Other retinal disorders	Anthropometric traits, Basal metabolic rate, Intelligence, Stroke
rs1016934	11	2.18E-12	*ELP4*	Strong meridian	Processed meat intake
rs618838	11	1.79E-09	*ACTN3*		Anthropometric traits, Impedance of body, Overall health rating, Red blood cell traits
rs116233906	11	9.33E-14	*SMIM38*		Heel bone mineral density
rs10737153	11	3.18E-09	*MYEOV*		Body mass, Breast cancer, Impedance of leg
rs12574286	11	5.77E-15	*GDPD4*		Hypertrophy of salivary gland, Platelet distribution width
rs1126809	11	2.56E-22	*TYR*	Eye colour, Foveal hypoplasia, Intraocular pressure, Ocular albinism, Oculocutaneous albinism, Optic disc size	Breast cancer, Hair colour, Hearing difficulties, Number of cancers, Skin cancer, Skin colour, Sunburn, Vitiligo
rs6483429	11	2.67E-09	*SESN3*		Hair/balding pattern

rs2080402	12	2.21E-12	*SLC6A13*	Age started wearing glasses	Comparative size at age 10, Lifetime number of sexual partners
rs3138142	12	5.58E-60	*RDH5*	Age started wearing glasses, Cataract, Macular thickness, Myopia, Pigmentary retinal dystrophy, Refractive error, Spherical power	Atrial fibrilation
rs76629482 *	12	3.38E-16	*NTN4*		
rs9796234 *	13	4.69E-18	*GRK1*		
rs28468687 *	14	1.02E-08	*RALGAPA1*		
rs1254260	14	1.18E-09	*LINC02322*	Age started wearing glasses, Glaucoma, Spherical power	Anthropometric traits, Basal metabolic rate, Forced expiratory volume, Heel bone mineral density, Impedance of legs, Menarche
rs1956524	14	2.13E-08	*RAD51B*		Asthma, Hand grip strength, Hayfever, allergic rhinitis or eczema, Male-pattern balding
rs10135971	14	4.47E-16	*ACTN1-AS1*	Macular degeneration, Spherical power	Exercises (swimming, cycling, keep fit, bowling), Forced vital capacity, Haematocrit percentage, Leg mass, Platelet traits, Pulse rate, Sensitivity/ hurt feelings
rs112145470 *	14	4.81E-16	*ZNF410*		
rs368205955	14	4.13E-46	*BBOF1*		Red cell distribution width
rs12147951 *	14	8.89E-112	*VSX2*		
rs1972565 *	14	5.23E-191	*VSX2*		
rs1972564 *	14	2.93E-74	*VSX2*		
rs118186707 *	14	1.70E-32	*VSX2*		
rs28488340 *	14	9.02E-31	*VSX2*		

rs888413	14	1.92E-14	*YLPM1*		Body fat percentage, Depression, Fed-up feelings, Guilty feelings, Monocyte count, Mood swings, Nervous feelings, Neuroticism, Platelet count, Worrier/anxious feelings
rs1800407	15	7.58E-25	*OCA2*	Age started wearing glasses, Cataract, Central retinal arteriolar equivalent, Central retinal vein equivalent, Cylindrical power, Eye colour, Macular thickness, Oculocutaneous albinism	Age of first facial hair, Hair colour, Skin cancer, Skin colour, Sunburn, Tanning
rs1648303 *	15	2.72E-08	*DUOX1*		
rs10083695	15	8.00E-13	*WDR72*		Haematocrit, Red blood cell count
rs3825991	15	2.70E-31	*RLBP1*		Age at menopause
rs1372613 *	15	1.31E-10	*LINS1*		
rs7206532	16	1.86E-09	*DYNLRB2*	Spherical power	
rs142963458	16	3.58E-18	*MEAK7*	Macular thickness	
rs1049868	16	1.91E-09	*GSE1*		Platelet traits
rs62064364	17	4.11E-16	*LINC02210-CRHR1*	Macular thickness	Alcohol consumption, Average total household income, Cereal type, Forced vital capacity, Hair/balding pattern, Hand grip strength, Height, Mean platelet volume, Mean reticulocyte volume, Mean sphered cell volume,Nap during day, Neuroticism, Oily fish intake, Plays computer games, Red blood cell traits, Relative age of first facial hair, White matter microstructre axial diusivities

rs4794029	17	1.95E-13	ABI3	Spherical power	Angina, Body fat, Coronary artery disease, Eosinophil traits, Forced expiratory volume, Height, High blood pressure, Impedance of limbs
rs56737642	17	2.87E-11	*FAAP100*	Age started wearing glasses, Catatract, Logmar, Spherical power	Ankle spacing width, Hair colour, Skin colour, Tanning
rs61586425 *	17	4.78E-12	*NPLOC4*		
rs62075724	17	2.91E-12	*TSPAN10*	Amblyopia, Cylindrical power, Logmar, Myopia, Spherical power	Hair colour, Tanning
rs7405453	17	2.24E-33	*TSPAN10*	Age started wearing glasses, Amblyopia, Astigmatism, Cylindrical power, Intraocular pressure, Logmar, Macular thickness, Spherical power	Ankle spacing width, Hair colour, Tanning
rs4800994	18	7.83E-10	*TCF4*		Frequency of stair climbing, Hot drink temperature, Length of time at current address, Number of days worked >10 mins, Works dayshift
rs1517034	18	3.90E-16	*RAX*		Eotaxin levels
rs17696543 *	18	2.93E-17	*CPLX4*		
rs76076446	19	1.07E-13	*RAX2*	Age-related macular degeneration, Cone-rod dystrophy, Macular thickness, Retinal dystrophy	
rs1232603	20	4.80E-12	*JAG1*		Birth weight, Haematocrit percentage, Haemoglobin concentration, Heel bone mineral density, Impedance of limbs, Red blood cell count

rs6077977	20	4.98E-19	*JAG1*		Heel bone mineral denisty, Height
rs8132685 *	21	3.58E-09	*C21orf62*		
rs2032576 *	22	4.80E-08	*MIATNB*		
rs5752638	22	1.63E-16	*MN1*		Cereal intake, Eosinophil count, Mean reticulocyte volume, Platelet traits, Pulse rate, Red blood cell count
rs5763593 *	22	1.01E-10	*MTMR3*		
rs2073946	22	5.17E-17	*LIF-AS1*		Heel bone mineral density, Immature reticulcyte fraction
rs75159625	22	2.83E-10	*WNT7B*	Macular thickness, Spherical power, Strong/weak meridian	Anthropometric traits, Contracture of palmar fascia, Haematocrit percentage, Red blood cell count
