# Supplementary material for: Sub-cellular level resolution of common genetic variation in the photoreceptor layer identifies continuum between rare disease and common variation
Source: PLoS Genet. 2023 Feb 27;19(2):e1010587. doi: 10.1371/journal.pgen.1010587 (PMC9997913; doi:10.1371/journal.pgen.1010587)
Supplement: S4 Table — Extended results of GWAS of the three photoreceptor cell layers, the outer nuclear layer (ONL), inner segment (IS) and outer segment (OS) as well as the results selected by meta-analysis using MTAG, labelled MTAG. Each locus found significantly associated following meta-analysis is listed alongside the chromosome (Chr) and location (BP). The reference and alternative alleles are listed alongside the allele frequency (AF) of the alternative allele (A1). The effect size refers to the effect of having an additional copy of the A1 allele and the standard error (SE) is further listed. (PDF) [file pgen.1010587.s009.pdf]

SNP	Chr	BP	A1	A2	AF	MTAG effect size	ONL effect size	OS effect size	IS effect size	MTAG p-value	ONL p-value	OS p-value	IS p-value	MTAG SE	ONL SE	OS SE	IS SE
rs112248193	1	3718402	A	G	0.13	-0.09	-0.26	-0.02	-0.09	2.73E-09	5.43E-04	0.60	2.73E-09	0.02	0.07	0.05	0.02
rs2128416	1	10700448	C	T	0.15	-0.53	-0.53	-0.02	-0.06	4.83E-13	4.83E-13	0.63	1.99E-04	0.07	0.07	0.04	0.02
rs11587687	1	110147801	A	G	0.44	0.18	-0.08	0.18	0.01	5.02E-09	0.12	5.02E-09	0.30	0.03	0.05	0.03	0.01
rs3790612	1	113084146	A	G	0.26	-0.27	-0.08	-0.27	-0.01	2.60E-15	0.17	2.60E-15	0.55	0.03	0.06	0.03	0.01
rs72683442	1	113468825	C	T	0.22	-0.35	-0.20	-0.35	-0.02	4.13E-21	1.18E-03	4.13E-21	0.16	0.04	0.06	0.04	0.01
rs410895	1	196898226	C	T	0.55	0.30	0.06	0.30	-0.01	8.19E-22	0.23	8.19E-22	0.17	0.03	0.05	0.03	0.01
rs6427827	1	200398387	A	T	0.62	0.42	0.42	-0.04	0.05	2.50E-16	2.50E-16	0.22	2.23E-06	0.05	0.05	0.03	0.01
rs11577827	1	202798831	T	C	0.53	0.37	0.37	0.07	0.05	2.77E-13	2.77E-13	0.03	6.56E-06	0.05	0.05	0.03	0.01
rs919655	1	214157972	A	G	0.12	0.46	0.46	2.03E-03	0.08	2.36E-09	2.36E-09	0.97	4.00E-07	0.08	0.08	0.05	0.02
rs12140498	1	222098690	T	C	0.20	0.37	0.37	0.08	0.05	3.14E-09	3.14E-09	0.04	2.89E-05	0.06	0.06	0.04	0.01
rs6426584	1	227374949	T	A	0.34	0.07	0.28	-0.03	0.07	7.03E-11	1.62E-07	0.36	7.03E-11	0.01	0.05	0.03	0.01
rs7594221	2	24112724	A	T	0.76	0.28	0.23	0.28	0.03	1.07E-14	8.19E-05	1.07E-14	0.01	0.04	0.06	0.04	0.01
rs116350483	2	145338686	T	C	0.02	-2.11	-2.11	-0.16	-0.33	2.10E-26	2.10E-26	0.19	2.86E-15	0.20	0.20	0.12	0.04
rs80265589	2	169027979	A	T	0.27	-0.42	-0.42	-0.14	-0.06	1.86E-13	1.86E-13	2.80E-05	4.21E-08	0.06	0.06	0.03	0.01
rs28416292	2	182507470	A	T	0.15	0.08	0.34	-0.02	0.08	1.78E-08	1.61E-06	0.61	1.78E-08	0.01	0.07	0.04	0.01
rs58172089	2	198916926	A	G	0.17	0.23	-0.07	0.23	-0.03	2.74E-08	0.32	2.74E-08	0.03	0.04	0.07	0.04	0.01
rs3755152	2	216822197	G	A	0.89	-0.31	0.42	-0.31	0.05	4.39E-10	5.15E-07	4.39E-10	1.83E-03	0.05	0.08	0.05	0.02
rs148388367	2	216850944	A	T	0.04	-1.30	-1.30	0.56	-0.14	1.20E-21	1.20E-21	9.63E-12	1.18E-06	0.14	0.14	0.08	0.03
rs201030469	2	218321984	G	A	0.14	-0.42	-0.42	-0.01	-0.07	6.58E-09	6.58E-09	0.82	1.30E-05	0.07	0.07	0.04	0.02
rs7564805	2	234228946	G	A	0.05	-0.78	-0.07	-0.78	0.01	6.16E-29	0.52	6.16E-29	0.55	0.07	0.12	0.07	0.02
rs34234056	3	14418444	G	A	0.51	0.17	0.01	0.17	-1.87E-03	2.37E-08	0.78	2.37E-08	0.86	0.03	0.05	0.03	0.01
rs11129176	3	25049310	A	G	0.28	0.32	0.32	0.02	0.04	1.53E-08	1.53E-08	0.60	1.31E-04	0.06	0.06	0.03	0.01
rs6775323	3	27721085	T	G	0.20	0.49	0.49	-0.04	0.08	5.79E-15	5.79E-15	0.34	4.35E-09	0.06	0.06	0.04	0.01
rs62282867	3	100972078	A	G	0.79	0.34	-0.16	0.34	-1.98E-03	4.63E-19	0.01	4.63E-19	0.88	0.04	0.06	0.04	0.01
rs111163508	3	129235423	C	G	0.16	-0.39	0.23	-0.39	0.03	2.28E-20	9.22E-04	2.28E-20	0.03	0.04	0.07	0.04	0.01
rs7430585	3	150112041	A	G	0.76	0.23	0.10	0.23	-0.05	1.26E-10	0.09	1.26E-10	2.66E-05	0.04	0.06	0.04	0.01
rs115237855	5	17186336	A	G	0.03	-0.68	0.17	-0.68	0.03	3.73E-12	0.29	3.73E-12	0.31	0.10	0.16	0.10	0.03
rs78303234	5	17218432	G	C	0.12	-0.26	-0.10	-0.26	0.01	3.32E-08	0.21	3.32E-08	0.58	0.05	0.08	0.05	0.02
rs30373	5	55745334	G	C	0.37	0.32	0.32	0.11	0.04	7.70E-10	7.70E-10	3.86E-04	4.10E-05	0.05	0.05	0.03	0.01
rs63338061	5	71486228	T	C	0.36	0.07	0.31	-7.24E-05	0.07	9.81E-11	3.71E-09	0.99	9.81E-11	0.01	0.05	0.03	0.01
rs17421627	5	87847586	G	T	0.07	1.15	1.15	0.16	0.17	3.30E-33	3.30E-33	0.01	4.57E-17	0.10	0.10	0.06	0.02
rs62391700	5	126093395	C	T	0.20	-0.45	-0.45	-0.13	-0.03	5.05E-13	5.05E-13	7.46E-04	0.04	0.06	0.06	0.04	0.01
rs1109114	5	148615946	T	C	0.43	0.37	0.37	-0.04	0.07	2.65E-13	2.65E-13	0.23	1.28E-10	0.05	0.05	0.03	0.01
rs1438692	5	148659664	A	G	0.42	0.07	0.32	-0.01	0.07	1.32E-11	3.59E-10	0.75	1.32E-11	0.01	0.05	0.03	0.01
rs6875105	5	173054917	C	T	0.38	-0.38	-0.38	-0.03	-0.03	1.39E-13	1.39E-13	0.27	4.48E-03	0.05	0.05	0.03	0.01
rs2326838	6	6901663	A	G	0.36	-0.23	-0.07	-0.23	-1.50E-03	7.86E-13	0.18	7.86E-13	0.89	0.03	0.05	0.03	0.01
rs12192672	6	7229619	A	G	0.30	-0.34	-0.34	0.01	-0.04	8.88E-10	8.88E-10	0.76	1.13E-04	0.06	0.06	0.03	0.01
rs17507554	6	11394287	A	G	0.05	0.69	0.69	-0.11	0.10	3.93E-09	3.93E-09	0.14	4.14E-05	0.12	0.12	0.07	0.02
rs6923949	6	35496366	G	A	0.82	0.23	0.36	0.23	0.04	4.22E-09	4.95E-08	4.22E-09	9.96E-04	0.04	0.07	0.04	0.01
rs375435	6	42661404	C	T	0.57	-0.25	-0.06	-0.25	-0.01	3.56E-16	0.20	3.56E-16	0.62	0.03	0.05	0.03	0.01
rs6910414	6	56726737	G	A	0.18	0.42	0.42	-0.16	-0.01	1.87E-10	1.87E-10	7.37E-05	0.67	0.07	0.07	0.04	0.01
rs947340	6	76747944	C	A	0.70	-0.25	-0.05	-0.25	0.03	1.17E-13	0.34	1.17E-13	0.02	0.03	0.06	0.03	0.01
rs74526772	6	106515218	A	T	0.04	-1.25	-1.25	-0.01	-0.18	1.13E-19	1.13E-19	0.90	6.24E-10	0.14	0.14	0.08	0.03
rs9639276	7	867033	T	C	0.17	0.41	0.41	-0.01	0.08	4.74E-10	4.74E-10	0.75	3.79E-09	0.07	0.07	0.04	0.01

rs12531825	7	8005174	A	G	0.12	0.45	0.45	0.09	0.08	5.94E-09	5.94E-09	0.07	2.34E-06	0.08	0.08	0.05	0.02
rs12719025	7	51100190	G	A	0.46	0.49	0.49	-3.73E-03	0.07	6.39E-22	6.39E-22	0.90	1.33E-10	0.05	0.05	0.03	0.01
rs111963714	7	99948655	G	T	0.21	0.27	0.20	0.27	0.03	5.96E-13	1.54E-03	5.96E-13	0.05	0.04	0.06	0.04	0.01
rs34926272	7	129591807	C	G	0.03	-1.05	-1.05	-0.15	-0.18	2.03E-11	2.03E-11	0.11	4.44E-08	0.16	0.16	0.10	0.03
rs62490856	8	10469030	A	G	0.13	0.33	-0.09	0.33	0.04	5.02E-13	0.25	5.02E-13	0.01	0.05	0.08	0.05	0.02
rs61675430	8	61671071	A	G	0.20	-0.41	-0.41	-7.74E-04	-0.06	1.30E-10	1.30E-10	0.98	1.33E-05	0.06	0.06	0.04	0.01
rs13263941	8	109121945	C	T	0.26	0.86	0.86	0.15	0.12	2.07E-50	2.07E-50	1.72E-05	6.09E-24	0.06	0.06	0.04	0.01
rs376067714	8	109141863	G	A	0.18	0.77	0.77	0.06	0.11	2.73E-24	2.73E-24	0.17	4.14E-12	0.08	0.08	0.05	0.02
rs9298817	9	21576591	C	A	0.66	-0.51	-0.51	-0.04	-0.04	2.90E-22	2.90E-22	0.21	6.77E-04	0.05	0.05	0.03	0.01
rs10781177	9	76593011	T	C	0.42	-0.29	-0.29	-0.01	-0.05	1.49E-08	1.49E-08	0.64	1.12E-05	0.05	0.05	0.03	0.01
rs717299	9	77185933	G	A	0.45	0.37	0.37	0.06	0.04	3.80E-13	3.80E-13	0.04	9.57E-05	0.05	0.05	0.03	0.01
rs111245635	10	48389841	T	C	0.02	1.26	1.26	0.30	0.23	8.41E-10	8.41E-10	0.02	1.27E-07	0.21	0.21	0.12	0.04
rs1947075	10	49741135	T	C	0.64	-0.32	-0.32	-0.07	-0.05	7.86E-10	7.86E-10	0.04	3.66E-06	0.05	0.05	0.03	0.01
rs7916697	10	69991853	G	A	0.76	-0.43	-0.43	-0.14	-0.06	3.80E-13	3.80E-13	1.40E-04	1.51E-07	0.06	0.06	0.04	0.01
rs11200922	10	85961758	G	A	0.46	0.33	0.08	0.33	-0.03	1.62E-26	0.12	1.62E-26	0.01	0.03	0.05	0.03	0.01
rs34309160	10	104034550	T	C	0.07	-0.53	-0.53	-0.04	-0.06	3.34E-08	3.34E-08	0.55	0.01	0.10	0.10	0.06	0.02
rs17102399	10	123435963	A	G	0.05	0.67	0.67	-0.02	0.07	2.44E-08	2.44E-08	0.75	3.95E-03	0.12	0.12	0.07	0.03
rs60401382	10	124227624	T	C	0.23	-0.30	0.07	-0.30	0.03	2.43E-16	0.27	2.43E-16	0.01	0.04	0.06	0.04	0.01
rs1016934	11	31720621	G	A	0.30	-0.39	-0.39	0.02	-0.03	2.18E-12	2.18E-12	0.46	0.01	0.06	0.06	0.03	0.01
rs618838	11	66328719	C	T	0.55	0.06	0.19	0.03	0.06	1.79E-09	1.49E-04	0.28	1.79E-09	0.01	0.05	0.03	0.01
rs116233906	11	68968271	A	C	0.04	-0.94	-0.94	-0.04	-0.11	9.33E-14	9.33E-14	0.59	3.09E-05	0.13	0.13	0.08	0.03
rs10737153	11	69281829	C	A	0.57	-0.30	-0.30	-0.02	-0.03	3.18E-09	3.18E-09	0.48	3.00E-03	0.05	0.05	0.03	0.01
rs12574286	11	76937602	C	G	0.21	-0.29	0.01	-0.29	0.01	5.77E-15	0.93	5.77E-15	0.41	0.04	0.06	0.04	0.01
rs1126809	11	89017961	A	G	0.30	-0.32	0.20	-0.32	0.04	2.56E-22	2.49E-04	2.56E-22	1.04E-03	0.03	0.05	0.03	0.01
rs6483429	11	95239787	T	C	0.46	-0.30	-0.30	8.18E-04	-0.04	2.67E-09	2.67E-09	0.98	4.72E-04	0.05	0.05	0.03	0.01
rs2080402	12	345175	C	T	0.56	0.22	-0.13	0.22	-0.03	2.21E-12	0.01	2.21E-12	3.18E-03	0.03	0.05	0.03	0.01
rs3138142	12	56115585	T	C	0.24	0.96	0.96	0.48	0.10	5.58E-60	5.58E-60	5.01E-41	7.10E-17	0.06	0.06	0.04	0.01
rs76629482	12	96178789	G	C	0.18	-0.53	-0.53	-0.06	-0.07	3.38E-16	3.38E-16	0.151	3.69E-07	0.07	0.07	0.04	0.01
rs9796234	13	114323997	C	T	0.52	0.27	0.19	0.27	0.03	4.69E-18	1.57E-04	4.69E-18	3.13E-03	0.03	0.05	0.03	0.01
rs28468687	14	36026342	A	G	0.18	0.38	0.38	0.06	0.05	1.02E-08	1.02E-08	0.13	2.01E-04	0.07	0.07	0.04	0.01
rs1254260	14	60835737	A	G	0.29	-0.20	0.25	-0.20	-0.03	1.18E-09	7.86E-06	1.18E-09	0.01	0.03	0.06	0.03	0.01
rs1956524	14	68800393	A	G	0.64	0.29	0.29	0.02	0.04	2.13E-08	2.13E-08	0.59	1.09E-03	0.05	0.05	0.03	0.01
rs10135971	14	69517494	A	G	0.34	0.43	0.43	-0.02	0.03	4.47E-16	4.47E-16	0.45	0.01	0.05	0.05	0.03	0.01
rs112145470	14	74356090	A	G	0.03	1.34	1.34	0.09	0.14	4.81E-16	4.81E-16	0.40	3.64E-05	0.17	0.17	0.10	0.03
rs368205955	14	74497636	G	T	0.02	-3.19	-3.19	-0.24	-0.50	4.13E-46	4.13E-46	0.08	1.56E-26	0.22	0.22	0.14	0.05
rs12147951	14	74642451	C	A	0.07	-2.34	-2.34	-0.10	-0.38	8.89E-112	8.89E-112	0.13	4.78E-67	0.10	0.10	0.06	0.02
rs1972565	14	74666824	G	A	0.82	1.91	1.91	0.11	0.30	5.23E-191	5.23E-191	0.01	7.95E-106	0.06	0.06	0.04	0.01
rs1972564	14	74666944	T	C	0.48	1.03	1.03	-0.04	0.15	2.93E-74	2.93E-74	0.22	1.40E-37	0.06	0.06	0.03	0.01
rs118186707	14	74686575	A	G	0.03	1.77	1.77	0.03	0.27	1.70E-32	1.70E-32	0.74	5.61E-18	0.15	0.15	0.09	0.03
rs28488340	14	74693803	G	C	0.40	-0.59	-0.59	-0.05	-0.08	9.02E-31	9.02E-31	0.14	1.07E-13	0.05	0.05	0.03	0.01
rs888413	14	75267396	T	C	0.47	0.39	0.39	0.07	0.07	1.92E-14	1.92E-14	0.02	3.64E-10	0.05	0.05	0.03	0.01
rs1800407	15	28230318	T	C	0.08	0.57	-0.03	0.57	-0.02	7.58E-25	0.70246515	7.58E-25	0.34	0.06	0.09	0.06	0.02
rs1648303	15	45445692	G	A	0.57	-0.28	-0.28	-0.01	-0.04	2.72E-08	2.72E-08	0.66	8.32E-04	0.05	0.05	0.03	0.01
rs10083695	15	53987147	G	A	0.49	-0.36	-0.36	-0.06	-0.06	8.00E-13	8.00E-13	0.03	5.67E-09	0.05	0.05	0.03	0.01
rs3825991	15	89761664	A	C	0.47	0.36	0.05	0.36	-0.04	2.70E-31	0.29	2.70E-31	1.10E-04	0.03	0.05	0.03	0.01
rs1372613	15	101204835	T	C	0.30	0.35	0.35	0.02	0.06	1.31E-10	1.31E-10	0.63	2.60E-07	0.05	0.05	0.03	0.01
rs7206532	16	80490131	C	T	0.52	0.30	0.30	0.02	0.03	1.86E-09	1.86E-09	0.56	3.06E-03	0.05	0.05	0.03	0.01

rs142963458	16	84561361	T	C	0.04	-0.73	0.77	-0.73	0.13	3.58E-18	3.55E-08	3.58E-18	4.56E-06	0.08	0.14	0.08	0.03
rs1049868	16	85706633	C	T	0.29	-0.34	-0.34	0.02	-0.05	1.91E-09	1.91E-09	0.61	3.52E-05	0.06	0.06	0.03	0.01
rs62064364	17	43654468	T	C	0.22	0.50	0.50	0.02	0.07	4.11E-16	4.11E-16	0.65	1.71E-07	0.06	0.06	0.04	0.01
rs4794029	17	47280301	C	T	0.68	0.24	0.06	0.24	-0.04	1.95E-13	0.27	1.95E-13	1.28E-04	0.03	0.05	0.03	0.01
rs56737642	17	79515509	G	A	0.48	-0.34	-0.34	0.21	-0.06	2.87E-11	2.87E-11	4.64E-11	3.90E-08	0.05	0.05	0.03	0.01
rs61586425	17	79590835	A	C	0.09	-0.48	-0.14	-0.48	-3.22E-03	4.78E-12	0.22	4.78E-12	0.89	0.07	0.11	0.07	0.02
rs62075724	17	79611410	C	T	0.46	0.38	0.38	-0.21	0.07	2.91E-12	2.91E-12	1.12E-10	1.94E-09	0.05	0.05	0.03	0.01
rs7405453	17	79615572	G	A	0.65	-0.39	0.40	-0.39	0.07	2.24E-33	4.81E-14	2.24E-33	3.40E-10	0.03	0.05	0.03	0.01
rs4800994	18	53403850	T	C	0.19	0.08	0.31	-0.12	0.08	7.83E-10	1.33E-06	1.88E-03	7.83E-10	0.01	0.06	0.04	0.01
rs1517034	18	56937489	A	G	0.31	0.45	0.45	-1.38E-03	0.07	3.90E-16	3.90E-16	0.97	9.89E-11	0.05	0.05	0.03	0.01
rs17696543	18	56971398	T	C	0.18	-0.56	-0.56	4.79E-03	-0.09	2.93E-17	2.93E-17	0.90	2.62E-10	0.07	0.07	0.04	0.01
rs76076446	19	3771586	A	G	0.02	-1.25	-1.25	0.14	-0.15	1.07E-13	1.07E-13	0.19	2.19E-05	0.17	0.17	0.10	0.04
rs1232603	20	10612963	T	C	0.33	-0.23	-4.45E-03	-0.23	0.01	4.80E-12	0.93	4.80E-12	0.56	0.03	0.05	0.03	0.01
rs6077977	20	10930708	G	A	0.49	0.27	-0.09	0.27	-0.01	4.98E-19	0.08	4.98E-19	0.28	0.03	0.05	0.03	0.01
rs8132685	21	34220618	T	C	0.52	-0.30	-0.30	-0.02	-0.04	3.58E-09	3.58E-09	0.54	1.01E-04	0.05	0.05	0.03	0.01
rs2032576	22	27089655	T	G	0.56	-0.28	-0.28	0.03	-0.04	4.80E-08	4.80E-08	0.41	1.19E-04	0.05	0.05	0.03	0.01
rs5752638	22	28188203	C	T	0.22	-0.51	-0.51	-0.09	-0.07	1.63E-16	1.63E-16	0.02	2.89E-08	0.06	0.06	0.04	0.01
rs5763593	22	30302238	C	T	0.63	0.34	0.34	-0.01	0.06	1.01E-10	1.01E-10	0.74	3.12E-09	0.05	0.05	0.03	0.01
rs2073946	22	30619599	A	G	0.31	0.46	0.46	0.07	0.08	5.17E-17	5.17E-17	0.04	2.82E-11	0.06	0.06	0.03	0.01
rs75159625	22	46377008	G	T	0.31	0.34	0.34	0.05	0.05	2.83E-10	2.83E-10	0.15	5.33E-05	0.05	0.05	0.03	0.01
