# Supplementary material for: Sub-cellular level resolution of common genetic variation in the photoreceptor layer identifies continuum between rare disease and common variation
Source: PLoS Genet. 2023 Feb 27;19(2):e1010587. doi: 10.1371/journal.pgen.1010587 (PMC9997913; doi:10.1371/journal.pgen.1010587)
Supplement: S5 Table — Several genes are involved in bringing about or shaping the photoreceptors’ electrical response to light (RHO, GNAT2, SAG, GRK1) or in recycling of the light-sensitive chromophore (ABCA4, RLBP1, RBP3, RDH5). Others are involved in maintaining outer retinal structure or in retinal and eye development. Superscript letters denote the relevant analyses in which the association was found: aGWAS; bdifferential association with ONL across concentric fields; cdifferential association with IS across concentric fields; ddifferential association with OS across concentric fields; eexome analysis. (PDF) [file pgen.1010587.s010.pdf]

| Gene | Location | Encoded Protein and Function | Mendelian disease | Mode of inheritance |
|---|---|---|---|---|
| ***Genes associated with rare monogenic retinal diseases*** | | | | |
| *CDHR1*[a,c] | 10q23.1 | Cadherin-related family member 1 (maintains rod and cone outer segment structure) | Cone-rod dystrophy; macular dystrophy | Recessive |
| *CERKL*[a] | 2q31.3 | Ceramide kinase-like protein (role in viability of cells with membranes rich in sphingolipids) | Rod-cone dystrophy; cone-rod dystrophy; widespread retinal degeneration | Recessive |
| *GNAT2*[a] | 1p13.3 | Cone-specific transducin (part of the phototransduction cascade in cone photoreceptors) | Achromatopsia (non-functioning cone photoreceptors) | Recessive |
| *GRK1*[a] | 13q34 | G-protein receptor kinase 1, rhodopsin kinase (phosphorylates activated rhodopsin to permit shut-off of the light response) | Oguchi disease, retinitis pigmentosa | Recessive |
| *IMPG1*[a] | 6q14.1 | Interphotoreceptor matrix proteoglycan 1 (role in retinal cell adhesion/ interphotoreceptor matrix) | Retinitis pigmentosa; vitelliform macular dystrophy | Dominant and recessive |
| *IMPG2*[a] | 3q12.3 | Interphotoreceptor matrix proteoglycan 2 (role in retinal cell adhesion/ interphotoreceptor matrix) | Retinitis pigmentosa; vitelliform macular dystrophy | Dominant and recessive |
| *JAG1*[a] | 20p12.2 | Jagged protein 1 (ligand for Notch proteins; function in cell-cell signalling) | Alagille syndrome | Dominant |
| *PRPH2*[a] | 6p21.1 | Peripherin-2 (important for rod and cone outer segment disc structure) | Macular pattern dystrophy; retinitis pigmentosa | Dominant and recessive |

| | | | | |
|---|---|---|---|---|
| *RAX2*[a,c] | 19p13.3 | Retina and anterior neural fold homeobox 2 transcription factor | Retinitis pigmentosa; cone-rod dystrophy | Dominant and recessive |
| *RBP3*[a] | 10q11.22 | Retinol binding protein 3 (role in retinoid recycling) | Retinal dystrophy associated with high myopia | Recessive |
| *RDH5*[a,b,d] | 14q24.1 | Retinol dehydrogenase 5 (role in retinoid recycling) | Fundus albipunctatus, retinitis pigmentosa | Recessive |
| *RHO*[a] | 3q22.1 | Rhodopsin (forms visual pigment in rods) | Retinitis pigmentosa | Dominant and recessive |
| *RLBP1*[a] | 15q26.1 | Retinaldehyde-binding protein 1 (role in retinoid recycling) | Bothnia dystrophy, retinitis pigmentosa | Recessive |
| *RP1L1*[a] | 15q26.1 | Retinitis pigmentosa 1-like protein 1 (role in photoreceptor connecting cilia) | Occult macular dystrophy; retinitis pigmentosa | Dominant and recessive |
| *SAG*[a] | 2q37.1 | S-antigen, arrestin (binds activated rhodopsin to permit shut-off of the light response) | Oguchi disease, retinitis pigmentosa | Recessive and dominant |
| *TULP1*[a] | 6p21.31 | Tubby-like protein 1 (role in rhodopsin transport within photoreceptors) | Retinitis pigmentosa; Leber congenital amaurosis | Recessive |
| *CFH*[d] | 1q31.3 | Complement factor H (regulates alternative complement pathway) | Early onset macular drusen | Dominant |
| *MERTK*[d] | 2q13 | c-mer protooncogene receptor tyrosine kinase (loss of function leads to defective phagocytosis of photoreceptor outer segments by RPE) | Retinitis pigmentosa; early onset retinal dystrophy | Recessive |

| | | | | |
|---|---|---|---|---|
| *ABCA4*[e] | 1p22.1 | ATP-binding cassette transporter A4 (functions as a flippase for N-retinylidene-phosphatidylenthanolamine; aids clearing of all-trans retinaldehyde) | Stargardt disease macular dystrophy; cone-rod dystrophy | Recessive |
| *NR2E3*[e] | 15q23 | Nuclear receptor subfamily 2 group E3 (transcription factor, role in rod photoreceptor development; loss of function leads to excess of S-cones) | Enhanced S-cone syndrome, Goldmann-Faver syndrome, retinitis pigmentosa | Recessive and dominant |
| *MYO7A*[e] | 11q13.5 | Myosin VIIA (motor protein functions in transport of opsin in photoreceptors and melanosomes in RPE) | Type 1 Usher syndrome (retinitis pigmentosa and deafness) | Recessive |
| ***Genes associated with oculocutaneous albinism (and foveal hypoplasia)*** | | | | |
| *OCA2*[a,e] | 15q12-15q13.1 | Oculocutaneous albinism 2 melanosomal transmembrane protein (protein regulates pH of melanosomes) | Oculocutaneous albinism | Recessive |
| *TYR*[a] | 11q14.3 | Tyrosinase (role in melanin synthesis) | Oculocutaneous albinism | Recessive |
| ***Genes associated with rare monogenic ocular maldevelopment (including microphthalmia)*** | | | | |
| *VSX2*[a,b] | 14q24.3 | Visual system homeobox 2 (role in eye development) | Microphthalmia | Recessive |
| *PITX2*[e] | 4q25 | Paired-like homeodomain transcription factor 2 (role in eye development) | Anterior segment dysgenesis; microphthalmia | Dominant |
| *ATOH7*[a] | 10q21.3 | Atonal basic helix-loop-helix transcription factor 7 (role in eye development) | Persistent primary hypoplastic vitreous; ocular maldevelopment, including microphthalmia | Recessive |

| | | | | |
|---|---|---|---|---|
| *CHD7*[a] | 8q12.2 | Chromodomain helicase DNA-binding protein 7 (role in control of neurocrest gene expression and in regulation of cel motility) | CHARGE syndrome | Dominant |
| *RARB*[a] | 3p24.2 | Retinoic acid receptor beta (role in eye and other organ development) | Microphthalmia, syndromic | Dominant and recessive |
| *RAX*[a] | 18q21.32 | Retina and anterior neural fold homeobox (role in eye development) | Microphthalmia | Recessive |
