# Supplementary material for: Sub-cellular level resolution of common genetic variation in the photoreceptor layer identifies continuum between rare disease and common variation
Source: PLoS Genet. 2023 Feb 27;19(2):e1010587. doi: 10.1371/journal.pgen.1010587 (PMC9997913; doi:10.1371/journal.pgen.1010587)
Supplement: S6 Table — List of genes associated with one of the three photoreceptor layers, the outer nuclear layer (ONL), inner segment (IS) and outer segment (OS) following gene burden testing. A significance threshold of P <5 × 10^-5^ is used. For each gene the gene name and chromosome is listed. Each gene is also annotated with any prior associations to ocular and non-ocular phenotypes. The number of individuals with the homozygous loss of function allele is listed as N. Two modes were tested, a missense (MS) model and a loss of function (LoF) model. (PDF) [file pgen.1010587.s011.pdf]

Gene Name	N	Chr	Effect Size	P-value	Layer	Model	Ocular phenotypes	Non-ocular phenotypes
TTK	26	6	-0.03	1.27E-05	ONL	MS	Macular thickness	Alcoholism, Basal metabolic rate, Blood pressure, Body mass, Breast cancer, Daytime sleep phenotypes, Erthrocytes, Heel bone mineral density, Height, Lung function, Pancreatic neoplasms
PGM5	21	9	-0.03	4.91E-05	ONL	MS		Bipolar disorder, Blood pressure, Chronic kidney disease, Creatinine levels, Estimated glomelular filtration rate, Leg mass, Metabolite levels, Urate levels
ABCA4	197	1	-0.03	4.34E-05	IS	MS	AMD, Cone-rod dystrophy, Retinitis pigmentosa, Refractive error, Stargardt disease	
PITX2	11	4	-0.03	4.67E-05	IS	MS	Axenfeld Anomaly, Glaucoma, Peters anomaly, Rieger Anomaly, Ring dermoid of Cornea	Acute appendicitis, Arrhythmia, Atrial fibrilation, Hypertension, Ischemic stroke
NR2E3	173	15	0.03	5.42E-06	IS	MS	Abnormality of the eye, Enhanced S-Cone syndrome, Goldmann-Favre syndrome, Macular thickness, Retinitis pigmentosa, Strong/weak meridian, Vertical cup-disc ratio	Adolescent idiopathic scoliosis, Chronic obstructive pulmonary disease, Height, Lung Function, Pulse pressure, Rate of cognitive decline in mild cognitive impairment, Red blood cell count, Urate levels
MYO7A	350	11	0.03	3.33E-05	OS	MS	Retinal dystrophy, Retinitis pigmentosa, Usher syndrome	Allergic disease, BMI, Deafness, Educational attainment, Hand grip strength, Height, Platelet traits, Ulcerative colitis
OCA2	480	15	0.04	2.33E-09	OS	MS	Age-related macular degeneration, Age started wearing glasses, Corneal astigmatism, Eye colour, Macular thickness, Oculocutaneous albinism, Refractive error	ADHD, Ease of Tanning, Educational attainment, Hair colour, Malignant neoplasm of skin, Skin colour, Sunburn, Vitiligo
GDPGP1	50	15	0.03	2.28E-05	OS	MS	Glaucoma, IOP	Allergic disease, Eczema, Heel bone mineral density, Height, Male-pattern baldness, Multiple sclerosis, Platelet traits, Type 2 diabetes, White blood cell traits
ABCD4	15	14	0.03	8.52E-06	IS	LoF	Glaucoma, IOP, Macular thickness, Refractive error, Spherical power	Body mass, Depression, Educational attainment, Height, Lung function, Methylmalonic aciduria and homocytinuria, Neuroticism, Red blood cell traits
CIB3	10	19	-0.03	2.69E-05	IS	LoF	Myopia	Heel bone mineral density, Height, Platelet traits, Urinary metabolites, White blood cell traits
