# Supplementary material for: Sub-cellular level resolution of common genetic variation in the photoreceptor layer identifies continuum between rare disease and common variation
Source: PLoS Genet. 2023 Feb 27;19(2):e1010587. doi: 10.1371/journal.pgen.1010587 (PMC9997913; doi:10.1371/journal.pgen.1010587)
Supplement: S7 Table — The results of geneset enrichment analysis using DEPICT applied to the GWAS results of the meta analysed PRC layers. All genesets which were significantly enriched following correction for false discovery rate are listed below. (PDF) [file pgen.1010587.s012.pdf]

Original gene set ID	Original gene set description	Nominal P-value	False discovery rate <5%
GO:0000978	RNA polymerase II core promoter proximal region sequence-specific DNA binding	5.65E-07	Yes
KEGG:Circadian Rhythm Mammal	KEGG:Circadian Rhythm Mammal	8.90E-07	Yes
ENSG00000134852	CLOCK subnetwork	1.76E-06	Yes
MP:0001303	abnormal lens morphology	1.78E-06	Yes
GO:0000981	sequence-specific DNA binding RNA polymerase II transcription factor activity	3.01E-06	Yes
MP:0002840	abnormal lens fiber morphology	9.06E-06	Yes
MP:0001304	cataracts	9.17E-06	Yes
GO:0051898	negative regulation of protein kinase B signaling cascade	9.81E-06	Yes
ENSG00000151623	NR3C2 subnetwork	1.16E-05	Yes
ENSG00000162992	NEUROD1 subnetwork	1.16E-05	Yes
ENSG00000100380	ST13 subnetwork	1.28E-05	Yes
ENSG00000164442	CITED2 subnetwork	1.57E-05	Yes
MP:0001299	abnormal eye distance/ position	1.63E-05	Yes
GO:0000987	core promoter proximal region sequence-specific DNA binding	1.93E-05	Yes
GO:0001159	core promoter proximal region DNA binding	2.86E-05	Yes
MP:0001328	disorganized retinal layers	2.87E-05	Yes
GO:0071478	cellular response to radiation	3.01E-05	Yes
GO:0000982	RNA polymerase II core promoter proximal region sequence-specific DNA binding transcription factor activity	3.22E-05	Yes
GO:0043401	steroid hormone mediated signaling pathway	3.53E-05	Yes
ENSG00000031544	ENSG00000031544 subnetwork	3.58E-05	Yes
ENSG00000160202	CRYAA subnetwork	4.02E-05	Yes
GO:0034339	regulation of transcription from RNA polymerase II promoter by nuclear hormone receptor	4.38E-05	Yes
GO:0001077	RNA polymerase II core promoter proximal region sequence-specific DNA binding transcription factor activity involved in positive regulation of transcription	4.97E-05	Yes

GO:0004879	ligand-activated sequence-specific DNA binding RNA polymerase II transcription factor activity	5.58E-05	Yes
REACTOME:Nuclear Receptor Transcription Pathway	REACTOME:Nuclear Receptor Transcription Pathway	5.68E-05	Yes
GO:0003707	steroid hormone receptor activity	7.87E-05	Yes
ENSG00000126351	THRA subnetwork	1.00E-04	Yes
GO:0071482	cellular response to light stimulus	1.00E-04	Yes
ENSG00000179588	ZFPM1 subnetwork	1.06E-04	Yes
ENSG00000151090	THRB subnetwork	1.08E-04	Yes
MP:0002864	abnormal ocular fundus morphology	1.18E-04	Yes
ENSG00000108094	CUL2 subnetwork	1.28E-04	Yes
MP:0001293	anophthalmia	1.30E-04	Yes
GO:0043491	protein kinase B signaling cascade	1.34E-04	Yes
ENSG00000124151	NCOA3 subnetwork	1.35E-04	Yes
GO:0004859	phospholipase inhibitor activity	1.42E-04	Yes
MP:0002944	increased lactate dehydrogenase level	1.44E-04	Yes
ENSG00000173486	FKBP2 subnetwork	1.53E-04	Yes
GO:0009416	response to light stimulus	1.57E-04	Yes
ENSG00000116016	EPAS1 subnetwork	1.63E-04	Yes
MP:0004362	cochlear hair cell degeneration	1.63E-04	Yes
ENSG00000206289	RXRB subnetwork	1.64E-04	Yes
ENSG00000206218	ENSG00000206218 subnetwork	1.64E-04	Yes
ENSG00000204231	RXRB subnetwork	1.64E-04	Yes
GO:0043010	camera-type eye development	1.78E-04	Yes
MP:0001382	abnormal nursing	1.90E-04	Yes
MP:0000784	forebrain hypoplasia	2.14E-04	Yes
ENSG00000004059	ARF5 subnetwork	2.20E-04	Yes
GO:0000976	transcription regulatory region sequence-specific DNA binding	2.23E-04	Yes
MP:0009434	paraparesis	2.25E-04	Yes
ENSG00000139323	POC1B subnetwork	2.26E-04	Yes
GO:0009314	response to radiation	2.33E-04	Yes
GO:0030032	lamellipodium assembly	2.42E-04	Yes
MP:0008511	thin retinal inner nuclear layer	2.53E-04	Yes
GO:0055102	lipase inhibitor activity	2.83E-04	Yes
GO:0001654	eye development	2.90E-04	Yes
GO:0005212	structural constituent of eye lens	2.96E-04	Yes
GO:0007030	Golgi organization	3.11E-04	Yes

GO:0001078	RNA polymerase II core promoter proximal region sequence-specificvDNA binding transcription factor activity involved in negative regulation of transcription	3.16E-04	Yes
MP:0005253	abnormal eye physiology	3.26E-04	Yes
ENSG00000132780	NASP subnetwork	3.40E-04	Yes
ENSG00000121671	CRY2 subnetwork	3.43E-04	Yes
ENSG00000173991	TCAP subnetwork	3.47E-04	Yes
MP:0005591	decreased vasodilation	3.47E-04	Yes
GO:0030275	LRR domain binding	3.53E-04	Yes
GO:0009755	hormone-mediated signaling pathway	3.54E-04	Yes
GO:0000977	RNA polymerase II regulatory region sequence-specific DNA binding	3.71E-04	Yes
ENSG00000113312	TTC1 subnetwork	3.90E-04	Yes
ENSG00000184357	HIST1H1B subnetwork	4.07E-04	Yes
MP:0002280	abnormal intercostal muscle morphology	4.14E-04	Yes
