# Supplementary material for: Sub-cellular level resolution of common genetic variation in the photoreceptor layer identifies continuum between rare disease and common variation
Source: PLoS Genet. 2023 Feb 27;19(2):e1010587. doi: 10.1371/journal.pgen.1010587 (PMC9997913; doi:10.1371/journal.pgen.1010587)
Supplement: S8 Table — The results of tissue enrichment analysis using DEPICT applied to the GWAS results of the meta analysed PRC layers. All Tissues which were nominally significantly enriched are listed below. (PDF) [file pgen.1010587.s013.pdf]

**MeSH term**	**MeSH first level term**	**MeSH second level term**	**Nominal P value**	**False discovery rate <5%**
A11.436	Epithelial Cells	Cells	1.17E-04	Yes
A09.371	Eye	Sense Organs	8.02E-04	Yes
A09.371.729	Retina	Sense Organs	1.64E-03	Yes
A10.615	Membranes	Tissues	4.41E-03	No
A10.615.550	Mucous Membrane	Tissues	0.01	No
A10.615.550.599	Mouth Mucosa	Tissues	0.02	No
A03.734.414	Islets of Langerhans	Digestive System	0.03	No
A14.549	Mouth	Stomatognathic System	0.04	No
A10.272	Epithelium	Tissues	0.04	No
A10.615.550.760	Respiratory Mucosa	Tissues	0.05	No
A04.531.520	Nasal Mucosa	Respiratory System	0.05	No
A09.531	Nose	Sense Organs	0.05	No
