# Supplementary material for: Sub-cellular level resolution of common genetic variation in the photoreceptor layer identifies continuum between rare disease and common variation
Source: PLoS Genet. 2023 Feb 27;19(2):e1010587. doi: 10.1371/journal.pgen.1010587 (PMC9997913; doi:10.1371/journal.pgen.1010587)
Supplement: S9 Table — List of SNPs with a significant z-score describing the differential effect on the ONL thickness at the foveal (F), intermediate (I) and peripheral (P) fields. The field (F1 or F2) and corresponding effect size from GWAS of thickness in each field are listed alongside the p-value of the comparative z-score. Each genetic variant is also annotated with associated gene and any ocular and non-ocular phenotypes previously associated with it. The different concentric comparisons are separated by bold horizontal lines. (PDF) [file pgen.1010587.s014.pdf]

SNP	Chr	F1	F2	F1 effect size	F2 effect size	P-value	Associated gene	Ocular phenotypes	General phenotypes
rs11708067	3	F	I	0.69	0.10	1.00E-07	*ADCY5*	Refractive error	Birth length, Birth weight, Cholesterol levels, Chronotype, Chronic kidney disease, Fasting blood glucose, Forced expiratory volume, Heel bone mineral density, Height, Type 2 diabetes
rs75757892	6	F	I	-1.12	-0.55	3.75E-06	*RREB1*	AMD, Cup-to-disc ratio, Refractive error	Birth weight, Blood glucose levels, Chronic kidney disease, Heel bone mineral density, Height, Hematocrit, Hemoglobin concentration, Impedance of body, Microcephaly, Mouth ulcers, Multiple sclerosis, Parkinsons, Red blood cell count, Self-reported math ability, Stroke, Type 2 diabetes, White blood cell count
rs45569432	14	F	I	-1.35	-2.17	3.27E-08	*VSX2*	Macular thickness, Refractive error	Cancer of endocrine glands, Chronotype, Red blood cell traits
rs1769287	1	F	P	-0.59	-0.05	6.90E-08	*SLC1A7*		Amyotrophic lateral sclerosis, Duration of fitness test, Height, Hippocampal atrophy, Lupus
rs6701735	1	F	P	0.67	-0.07	5.14E-07	*MIR29B2CHG*		Pulse rate, White blood cell count
rs11125573	2	F	P	0.54	0.08	2.27E-06	*CCDC88A*		Appendicular lean mass

rs11720108	3	F	P	0.71	-0.04	9.67E-13	*ADCY5*	Refractive error	Birth weight, Chronic kidney disease, Chronotype, Forced expiratory volume, Heel bone mineral density, Sex hormone-binding globulin levels, Statin medication, Type 2 diabetes
rs55914544	5	F	P	0.65	-0.04	2.53E-08	*WWC1*		Blood pressure, BMI, Body fat, Cerebellum cortex volume, Epithelial ovarian cancer
rs75757892	6	F	P	-1.12	-0.20	1.84E-15	*RREB1*	AMD, Cup-to-disc ratio, Refractive error	Birth weight, Blood glucose levels, Chronic kidney disease, Heel bone mineral density, Height, Hematocrit, Hemoglobin concentration, Impedance of body, Microcephaly, Mouth ulcers, Multiple sclerosis, Parkinsons, Red blood cell count, Self-reported math ability, Stroke, Type 2 diabetes, White blood cell count
6:150016812_GA_G	6	F	P	0.61	0.13	1.54E-06			
rs746173658	8	F	P	0.22	0.93	4.85E-12			
rs1337805	9	F	P	-0.52	0.06	2.07E-07	*PTPRD*	Myopia	ADHD, Anxiety, Asthma, Blood pressure, Cancer, Cholesterol, Coronary disease, Depression, Diabetes, Epilepsy, Forced exipratory volume, Heel bone mineral density, Height, Menarche

rs11594394	10	F	P	0.61	0.04	1.45E-08	*FRMPD2*	Age started wearing glasses, Myopia, Refractive error, Spherical power	
rs12225226	11	F	P	0.20	-0.45	2.33E-07	*LINC01488*		BMI, Breast cancer, Cardiomyopathy, Height, Monocyte count, White blood cell count
rs3138142	12	F	P	1.37	0.81	1.03E-07	*RDH5*	Age started wearing glasses, Cataract, Early AMD, Fundus albipunctatus, Hypermetropia, Macular thickness, Myopia, Pigmentary retinal dystrophy, Refractive error, Retinal dystrophy, Retinitis pigmentosa, Spherical power	
rs3921811	12	F	P	0.57	-0.02	7.37E-10	*PLEKHA5*		Time spent outdoors in winter
rs1493529	13	F	P	-0.48	0.07	1.05E-08	*ENOX1*		Arteries, Blood pressure, Depression, End-stage coagulation, Glucose, Mental health disorders, Prostate tumour
rs1972565	14	F	P	1.27	1.94	8.59E-09	*VSX2*	Macular thickness, Refractive error	Blood pressure, Chronotype, Coronary artery disease

rs3138142	12	I	P	1.28	0.81	1.31E-07	*RDH5*	Age started wearing glasses, Cataract, Early AMD, Fundus albipunctatus, Hypermetropia, Macular thickness, Myopia, Pigmentary retinal dystrophy, Refractive error, Retinal dystrophy, Retinitis pigmentosa, Spherical power	
