# Supplementary material for: Sub-cellular level resolution of common genetic variation in the photoreceptor layer identifies continuum between rare disease and common variation
Source: PLoS Genet. 2023 Feb 27;19(2):e1010587. doi: 10.1371/journal.pgen.1010587 (PMC9997913; doi:10.1371/journal.pgen.1010587)
Supplement: S10 Table — List of SNPs with a significant z-score describing the differential effect on the IS thickness at the foveal (F), intermediate (I) and peripheral (P) fields. The field (F1 or F2) and corresponding effect size from GWAS of thickness in each field are listed alongside the p-value of the comparative z-score. Each genetic variant is also annotated with associated gene and any ocular and non-ocular phenotypes previously associated with it. The different concentric comparisons are separated by bold horizontal lines. (PDF) [file pgen.1010587.s015.pdf]

SNP	Chr	F1	F2	F1 effect size	F2 effect size	P value	Associated gene	Ocular phenotypes	General phenotypes
rs4721061	7	F	I	-0.23	-0.09	3.40E-11	*TMEM106B*		Coronary artery disease, Dementia, Depression, Height, Irritability, Mood swings, Neuroticism
rs55798570	10	F	I	0.13	1.61E-03	2.06E-06	*CDHR1*	Cone-rod dystrophy, Refractive error, Retinitis pigmentosa	
rs61773269	1	F	P	-0.23	-0.03	1.43E-06	LRRC8D		
rs7430585	3	F	P	-0.26	-0.05	1.27E-18	*TSC22D2*	Age started wearing glasses	Blood pressure, Brain morphology, Cholesterol, Platelet count, Pulse pressure, Type 2 diabetes
rs59515506	4	F	P	-0.34	-0.01	7.15E-09	*RUFY3*		Cardiovascular disease, Hippocampus, Parkinson disease
rs4131080	5	F	P	-0.12	-0.02	1.95E-06	*HNRNPH1*		Neutrophil count, White blood cell count
5:71646122_AATTT_A	5	F	P	-0.03	0.08	4.20E-07			
6:31806799_CA_C	6	F	P	-0.13	0.01	3.33E-08			
rs13237518	7	F	P	-0.23	-0.02	1.77E-26	*TMEM106B*		Coronary artery disease, Dementia, Depression, Height, Irritability, Mood swings, Neuroticism, Type 2 diabetes

rs141629142	9	F	P	0.40	0.06	9.15E-06	*RORB*	Advanced AMD, Macular thickness, Myopia, Refractive error	Alcohol consumption, Blood pressure, BMI, Cholesterol, Chronotype, Creatinine, Epilepsy, Erythrocytes, Glomeluar filtration rate, Glucose, Heart failure, Height
rs1409396	10	F	P	0.13	0.02	7.63E-09	*PIP4K2A*		Frequency of walking for pleasure, Height, Platelet distribution width
rs55798570	10	F	P	0.13	-0.06	1.12E-13	*CDHR1*	Cone-rod dystrophy, Refractive error, Retinitis pigmentosa	
rs200916002	14	F	P	-0.07	-0.21	1.46E-06	*BBOF1*		
rs55634267	17	F	P	0.20	0.05	1.08E-11	*GNGT2*	Refractive error, Spherical power	Angina, Asthma, Chronic lower respiratory disease, Coronary artery disease, Eosinophil counts, Hypertension, Impedance of limbs, Metabolite errors, Prostate cancer
rs850526	17	F	P	-0.16	-0.04	7.56E-08	*GNGT2*	Spherical power, Refractive error	Appendicular lean mass, Asthma, Chronic lower respiratory disease, Metabolite levels, Primary sclerosing cholangitis, Prostate cancer
rs76076446	19	F	P	0.31	-0.09	1.76E-09	*RAX2*	AMD, Cone-rod dystrophy, Macular thickness, Retinal dystrophy	

rs2236665	21	F	P	0.16	0.01	1.53E-13	*RRP1B*	Refractive error, Spherical power	Blood pressure, Coronary artery disease, Haemoglobin traits, High blood pressure, Hypertension, Red blood cell traits
rs1041451	21	F	P	-0.14	-0.01	2.74E-10	*C2CD2*		
rs6414375	3	I	P	-0.18	-0.06	9.14E-10	*TSC22D2*	Age started wearing glasses, Glaucoma, IOP, Macular thickness, Refractive error	Blood pressure, Brain morphology, Cholesterol, Platelet count, Pulse pressure, Type 2 diabetes
rs13171669	5	I	P	-0.01	0.07	3.06E-06	*AFAP1L1*	Macular thickness	Blood pressure, BMI, Forced expiratory volume, Glomelular filtration, Hearing difficulties, Hypertension, Socioeconomic factors
rs11974335	7	I	P	-0.09	-0.01	1.15E-05	*TMEM106B*		Dementia, Depression, Height, Impedance of body, Irritability, Mood swings, Neuroticism
21:45115055_CT_C	21	I	P	0.11	0.01	8.27E-08			
