# Supplementary material for: Sub-cellular level resolution of common genetic variation in the photoreceptor layer identifies continuum between rare disease and common variation
Source: PLoS Genet. 2023 Feb 27;19(2):e1010587. doi: 10.1371/journal.pgen.1010587 (PMC9997913; doi:10.1371/journal.pgen.1010587)
Supplement: S11 Table — List of SNPs with a significant z-score describing the differential effect on the OS thickness at the foveal (F), intermediate (I) and peripheral (P) fields. The field (F1 or F2) and corresponding effect size from GWAS of thickness in each field are listed alongside the Bonferroni adjusted p-value of the comparative z-score. Each genetic variant is also annotated with associated gene and any ocular and non-ocular phenotypes previously associated it. The different concentric comparisons are separated by bold horizontal lines. (PDF) [file pgen.1010587.s016.pdf]

SNP	Chr	F1	F2	F1 effect size	F2 effect size	P value	Associated gene	Ocular phenotypes	General phenotypes
rs1329428	1	F	P	0.64	0.24	4.79E-13	*CFH*	Advanced AMD, Chronic central serous retinopathy, Disorders of the choroid and retina	Lung function
rs200857348	2	F	P	1.45	0.60	3.96E-10	*MREG*	Macular thickness	Albuminuria, Breast neoplasms, Coronary artery disease, Heart failure, Socioeconomic factors
rs869016	2	F	P	-0.31	0.02	3.57E-09	*MERTK*	Retinitis pigmentosa	Blood pressure, Coronary artery disease, Heel bone mineral density, Multiple sclerosis
rs556679	6	F	P	0.56	0.18	8.11E-06	*C2*	AMD, Diabetic retinopathy, Other retinal disorders	Ankle spacing width, Asthma, Autoimmune disease, Body fat, Coeliac disease, Coffee consumption, Glomelular filtration rate, Membranous nephropathy, Multiple sclerosis, Psoriasis, Type 1 diabetes
rs199637836	10	F	P	-0.76	-0.24	1.38E-14			
rs3138141	12	F	P	0.76	0.32	4.48E-13	*RDH5*	Advanced AMD, Age started wearing glasses, Cataract, Fundus albipunctatus, Hypermetropia, Macular thickness, Myopia, Retinal dystrophy, Spherical power	Atrial fibrilation
rs62125245	19	F	P	0.44	0.11	7.07E-06	*NXNL1*		
rs3138142	12	I	P	0.78	0.32	3.08E-17	*RDH5*	Advanced AMD, Age started wearing glasses, Cataract, Fundus albipunctatus, Hypermetropia, Macular thickness, Myopia, Retinal dystrophy, Spherical power	Atrial fibrillation
