# Supplementary material for: Sub-cellular level resolution of common genetic variation in the photoreceptor layer identifies continuum between rare disease and common variation
Source: PLoS Genet. 2023 Feb 27;19(2):e1010587. doi: 10.1371/journal.pgen.1010587 (PMC9997913; doi:10.1371/journal.pgen.1010587)
Supplement: S1 Text — (PDF) [file pgen.1010587.s017.pdf]

The UK Biobank Eye and Vision Consortium members are:

Naomi Allen, Tariq Aslam, Denize Atan, Sarah Barman, Jenny Barrett, Paul Bishop, Graeme Black, Tasanee Braithwaite, Roxana Carare, Usha Chakravarthy, Michelle Chan, Sharon Chua, Alexander Day, Parul Desai, Bal Dhillon, Andrew Dick, Alexander Doney, Cathy Egan, Sarah Ennis, Paul Foster, Marcus Fruttiger, John Gallacher, David Garway-Heath, Jane Gibson, Jeremy Guggenheim, Chris Hammond, Alison Hardcastle, Simon Harding, Ruth Hogg, Pirro Hysi, Pearse Keane, Peng Tee Khaw, Anthony Khawaja, Gerassimos Lascaratos, Thomas Littlejohns, Andrew Lotery, Phil Luthert, Tom MacGillivray, Sarah Mackie, Bernadette McGuinness, Gareth McKay, Martin McKibbin, Tony Moore, James Morgan, Richard Oram, Eoin O'Sullivan, Chris Owen, Praveen Patel, Euan Paterson, Tunde Peto, Axel Petzold, Nikolas Pontikos, Jugnoo Rahi, Alicja Rudnicka, Naveed Sattar, Jay Self, Panagiotis Sergouniotis, Sobha Sivaprasad, David Steel, Irene Stratton, Nicholas Strouthidis, Cathie Sudlow, Zihan Sun, Robyn Tapp, Dhanes Thomas, Emanuele Trucco, Adnan Tufail, Ananth Viswanathan, Veronique Vitart, Mike Weedon, Katie Williams, Cathy Williams, Jayne Woodside, Max Yates, Jennifer Yip, Yalin Zheng.
